# Enhancing G-quadruplex-based DNA nanotechnology: new lipophilic DNA G-quadruplexes with TBDPS modifications†

**DOI:** 10.1039/d5ra01033k

**Published:** 2025-05-29

**Authors:** Maria Marzano, Maria Grazia Nolli, Stefano D'Errico, Andrea Patrizia Falanga, Monica Terracciano, Principia Dardano, Luca De Stefano, Gennaro Piccialli, Nicola Borbone, Giorgia Oliviero

**Affiliations:** a Dipartimento di Farmacia, Università degli Studi di Napoli Federico II Via Domenico Montesano 49 80131 Napoli Italy stefano.derrico@unina.it nicola.borbone@unina.it; b Institute of Applied Sciences and Intelligent Systems “Eduardo Caianiello”, Unit of Naples, National Research Council Naples 80131 Italy; c ISBE-IT, Università degli Studi di Napoli Federico II 80138 Napoli Italy; d Dipartimento di Medicina Molecolare e Biotecnologie Mediche, Università degli Studi di Napoli Federico II Via Sergio Pansini 5 80131 Napoli Italy

## Abstract

This study introduces a novel class of highly lipophilic DNA G-quadruplexes (G4s) obtained by installing the lipophilic *tert*-butyldiphenylsilyl group (TBDPS) at both ends of 5′-CG_*n*_-3′-3′-G_*n*_C-5′ oligonucleotides (ONs), where *n* = 1 or 2, featuring a 3′-3′ inversion of polarity, thus obtaining symmetric (TBDPS-5′-CG_*n*_-3′-3′-G_*n*_C-5′-TBDPS)_4_ lipophilic G4s after annealing in K^+^-containing buffer. The new 5′-bis-conjugated TBDPS-ONs were synthesized using a tailored solid-phase approach, where the first nucleoside (dC) was linked to the polymeric support *via* the exocyclic amino group of the nucleobase. The effect of the presence of the TBDPS groups on G4 formation, stability, and propensity to form supramolecular G4 aggregates was assessed using ^1^H NMR, circular dichroism (CD), polyacrylamide gel electrophoresis (PAGE), scanning electron microscopy (SEM), dynamic light scattering (DLS), and atomic force microscopy (AFM) analyses. The results demonstrate that the presence of four TBDPS groups at the 5′-ends of the G4 strands enhances the stability of the G4s, enabling their formation even at low K^+^ concentration (20 mmol L^−1^). We report the formation of the smallest tetramolecular G4 observed to date, [(TBDPS-5′-CG-3′-3′-GC-5′-TBDPS)_4_], which contains only two G-tetrads. Notably, this structure did not form when using the corresponding oligonucleotide sequence lacking the TBDPS groups, even at high K^+^ concentrations (up to 1 mol L^−1^). Furthermore, the lipophilic shells located at the 5′-faces of the G4 structures promote the formation of submicrometric coffee bean-like aggregates composed of G4 units. These novel lipophilic G4s exhibit two key features: high structural symmetry and a tunable balance between their lipophilic (TBDPS groups) and hydrophilic (oligonucleotide strands) moieties. This tunability allows for precise modulation of both the extent and the properties of the resulting supramolecular assemblies. These findings provide valuable insights into developing G4-based systems in DNA nanotechnology.

## Abstract 2

The ON 1, bearing the lipophilic TBDPS groups at both 5′ ends, produces − after the annealing procedure in K^+^-containing buffer − the highly lipophilic G-quadruplex 2, which spontaneously assembles into ordered G4 aggregates.

## Graphical abstract

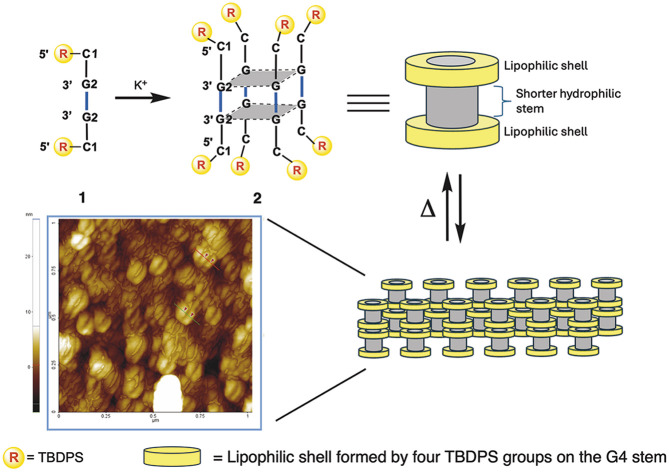

## Introduction

Recently, the use of short DNA and RNA strands has grown significantly, driven by their crucial biological roles, pharmacological activities, and their capacity to form predictable supramolecular structures *via* a bottom-up self-assembly process starting from relatively small oligonucleotides (ONs).^1–6^ This latter aspect, defined as DNA nanotechnology, exploits the well-known self-assembly properties of ONs to create well-defined supramolecular architectures with promising applications in several fields, such as the development of novel biomaterials,^7,8^ nanodevices,^9–13^ and bioelectronics.^14–16^ A technique known as DNA origami utilizes ONs with precisely designed base sequences to construct three-dimensional DNA nanostructures.^17–21^ These nanostructures can serve as valuable macromolecules for specific nanotechnology applications or as intriguing objects with unique shapes, never realized at this scale.

Current research in DNA-based therapeutics and nanotechnology increasingly focuses on DNA-conjugated hybrid materials, where diverse functional groups, such as lipophilic,^22–24^ cationic, or polymeric groups,^25,26^ are attached to ON fragments. Lipophilic groups of varying sizes have been conjugated with ONs to enhance specific properties. Lipophilic-ONs (LONs) have demonstrated enhanced cellular uptake and stability in antisense therapy, resulting in more efficient gene silencing.^27,28^ LONs, as aptamers, exhibit high specificity and affinity for target proteins, enabling their use in targeted drug delivery and diagnostics.^24,29–31^ LONs can also exhibit interesting self-aggregation properties through lipophilic attraction and specific recognition of DNA bases (Watson–Crick and Hoogsteen base pairing). These properties enable LONs to aggregate in aqueous solutions, forming micellar particles well-suited for cell endocytosis and other unprecedented supramolecular architectures.^26^ The conjugation of an ON with lipophilic groups can enhance its cellular uptake by providing an additional anchor for membrane interaction. Moreover, LONs have demonstrated the ability to form transmembrane channels when organized in a G-quadruplex (G4) structure, facilitating ion transport and offering innovative approaches for biochemical and pharmacological research.^32,33^

**Fig. 1:**
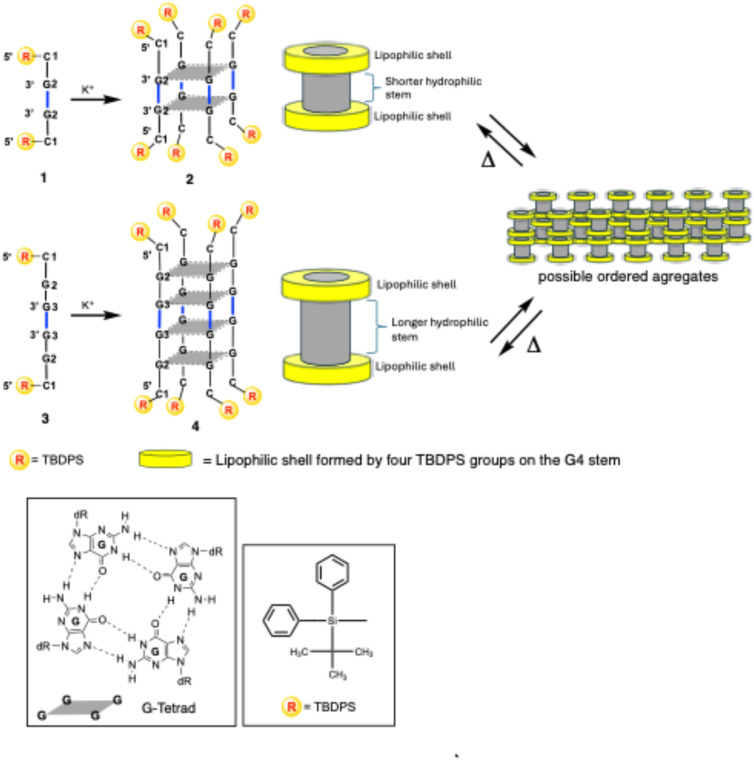
Structures of G4s 2 and 4 carrying eight 5′-ending TBDPS groups obtained by annealing of ONs 1 and 3, respectively. A tentative structural organization of G4 building blocks into ordered G4 aggregates through lipophilic interactions is depicted on the right. Inset: G-tetrad and TBDPS structures.

Among the secondary structures of DNA and RNA, the G4s formed by guanine (G)-rich sequences play essential biological roles in cells. They are remarkably versatile for applications in supramolecular chemistry and in the development of innovative biomaterials, biosensors, and bioelectronics.^9,34–39^ The unique properties of G4s arise from their ability to self-assemble, display extensive polymorphism, and offer tunable stability.^40,41^ G4 topology and stability can be fine-tuned by varying the sequence and length of the G-rich ONs and the nature and concentration of stabilizing cations.^42–44^ The binding motif of a G4 is the G-tetrad, formed by the coplanar arrangement of four G bases belonging to one, two, or four G-rich ONs mutually linked by eight Hoogsteen's hydrogen bonds (Fig. 1). Two or more G-tetrads stabilize the entire G4 structure through their reciprocal π–π stacking interactions and by coordinating monovalent cations located in the center of each G-tetrad (*e.g.*, Na^+^) or between two adjacent G-tetrads (*e.g.*, K^+^), depending on the specific cations' radius. Thanks to the octahedral coordination with carbonyl oxygen atoms belonging to two adjacent G-tetrads, potassium-coordinated G4s are more stable than the corresponding sodium-coordinated G4s. Moreover, it is well known that the incubation with sodium ions favors the formation of antiparallel G4 topologies, whereas that with potassium ions drives towards the obtainment of parallel G4 assemblies and favors the formation of supramolecular G4 aggregates.^44^ The G4s′ structural features typically render G4s more stable than DNA duplexes of equivalent length, enabling the construction of the supramolecular structures known as G-wires.^45,46^ A few examples of G4 structures formed by G-rich LONs have been reported in the literature. One notable case describes a lipophilic G4 used as a transmembrane channel, incorporating lipophilic groups within the ON sequences or at their 3′ ends – specifically, a C12 carbon linker^47^ or a cholesteryl moiety,^48^ respectively. The monomolecular thrombin binding aptamer (TBA) G-quadruplex has also been stabilized through 5′- and 3′-end functionalization with various lipophilic polyaromatic groups.^49^ Finally, numerous studies have explored the role of lipophilic substituents on guanosine derivatives in promoting the formation of G-quadruplex supramolecular aggregates.^50,51^

This study introduces a novel class of highly lipophilic G4 complexes (2 and 4, Fig. 1), each bearing eight *tert-*butyldiphenylsilyl (TBDPS) groups. It explores their enhanced stability and ability to form supramolecular assemblies with amphiphilic properties, governed by the structural features of the G4 building blocks. These G4 structures exhibit two key attributes: high symmetry and a tunable ratio between their lipophilic (TBDPS groups) and hydrophilic (ON strands) moieties. This tunability enables precise control over the extent and the properties of the resulting supramolecular assemblies. The G4s 2 and 4 were obtained by annealing the G-rich ONs 1 and 3, namely d(TBDPS-5′-CG_*n*_-3′-3′-G_*n*_C-5′-TBDPS) with *n* = 1 or 2, respectively. These sequences incorporate a 3′-3′ inversion of polarity and feature two lipophilic TBDPS groups conjugated to the two 5′-ends, resulting in symmetric ONs. The conjugation of ONs with TBDPS groups has long been a widely employed strategy in synthesizing nucleosides and ONs, effectively protecting the 5′ and 3′ sugar positions. Additionally, TBDPS-modified ONs have been utilized to enhance lipophilicity, thereby improving the stability of duplex and G-quadruplex structures.^31^

**Fig. 2:**
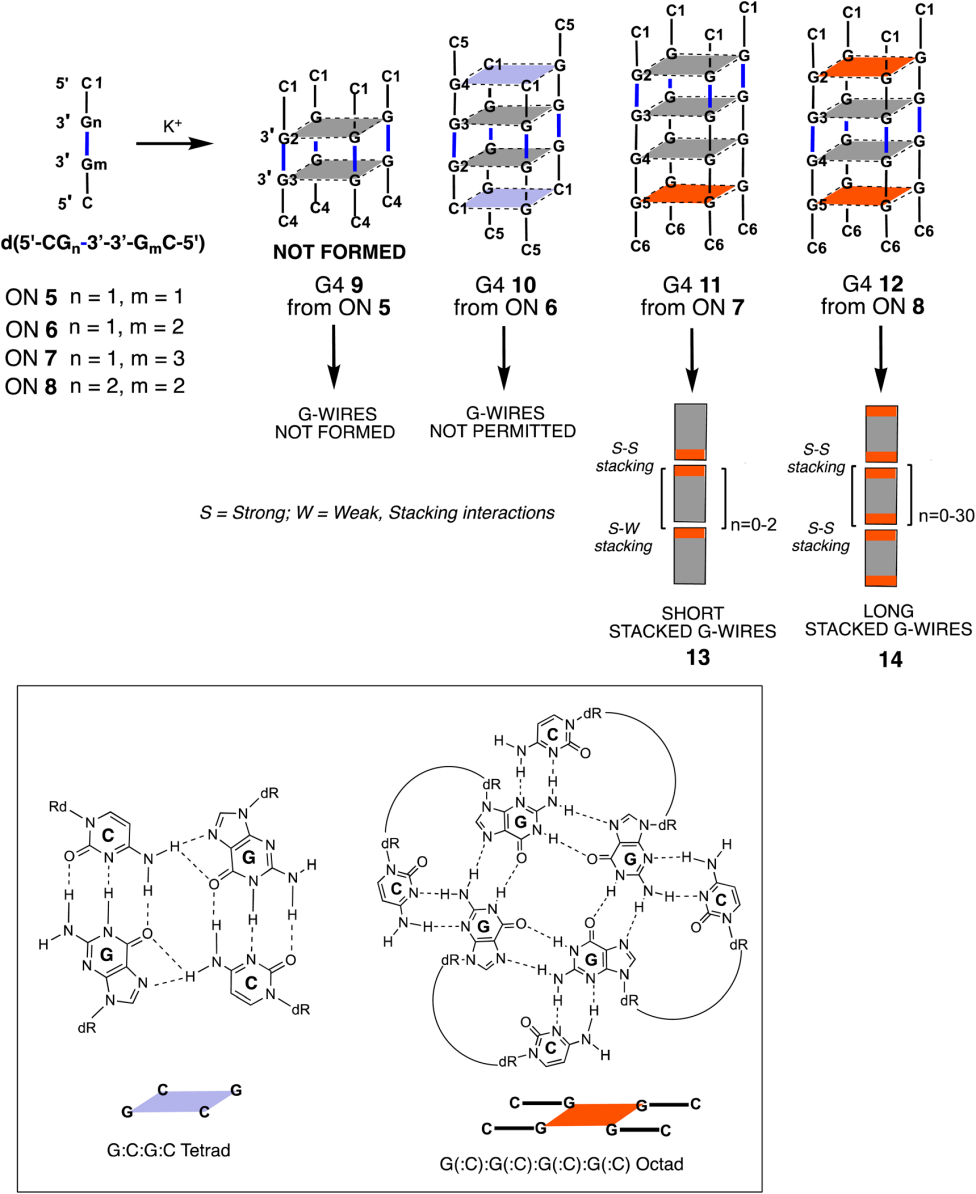
Schematic representation of G4s formed by ONs 5–8 and their ability to form G-wire assemblies. The 5′-ending G-tetrads involved in the formation of the G(:C):G(:C):G(:C):G(:C) octad and π–π stacking interactions between G4 units are represented in red.

In a previous study, our research group investigated non-natural G-rich ONs incorporating a 3′-3′ inversion of polarity, namely d(5′-CG_*n*_-3′-3′-G_m_C-5′) (5–8, Fig. 2).^52^ We explored their capacity to form G4 structures and supramolecular assemblies, called stacked G-wires, through the multimerization of G4 building blocks (13 and 14). The multimerization process occurs *via* π–π stacking interactions between different G4 units, further stabilized by the formation of planar G(:C):G(:C):G(:C):G(:C) octads at each 5′-end of the G4 monomer (highlighted in red in Fig. 2).^53,54^ Notably, the short ON 5 did not form the tetramolecular G4 9, likely because of the insufficient stabilization achievable by the π–π stacking of only two G-tetrads. In contrast, the ON 8 successfully formed the G4 monomer 12 and its corresponding supramolecular G-wire assembly 14 thanks to the presence of π–π stacking between octads (in red in Fig. 2).

Herein, we reported on the formation of the new lipophilic G4 4 and the unprecedented smallest tetramolecular G4 2, exploiting the stabilizing effect of eight TBDPS groups installed at both the 5′ ends of ONs 1 and 3. Furthermore, we examined the aggregation properties of these lipophilic G4s in constructing supramolecular assemblies, observing that the aggregate extension could be modulated by varying the ratios between their lipophilic shell (TBDPS groups) and hydrophilic moieties (length of ON strands).

## Results and discussion

### Preparation of ONs 1 and 3 and G4s 2 and 4

**Scheme 1:**
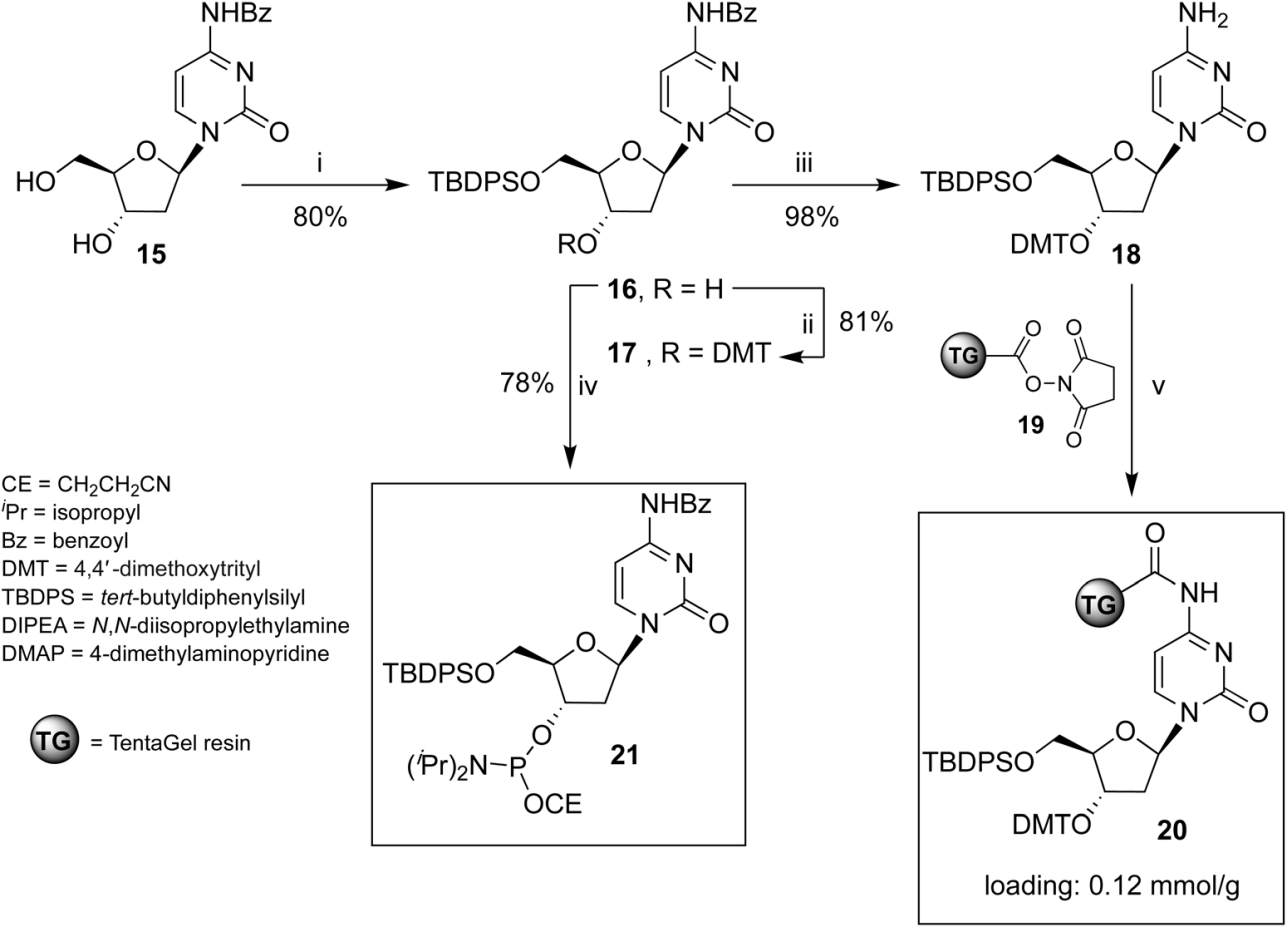
Reagents and conditions: (i) TBDPSCl, imidazole, pyridine, r.t., 4 h;^57^ (ii) DMTCl, DMAP, pyridine, 40 °C, 16 h; (iii) NH_3_ in CH_3_OH, r.t., 2 h; (iv) (^i^Pr)_2_NP(Cl)OCE, DIPEA, CH_2_Cl_2_, r.t., 2 h; (v) 19, CH_2_Cl_2_, r.t., 16 h.

Oligonucleotides 1 and 3 bearing two TBDPS groups at their 5′-ends were synthesized using the solid-phase phosphoramidite method. This process required the preparation of the solid support 20 (Scheme 1), in which the 5′-O-TBDPS-3′-O-DMT-2′-deoxycytidine 18 was bonded to the TentaGel (TG) support through the exocyclic amino group of the base. This functionalization strategy^55,56^ produced support 20 and enabled the synthesis of the 5′-labelled ONs, thereby expanding the range of obtainable ON structures.

### Preparation of solid support 20

The unprecedented new support 20 was obtained by reacting deoxycytidine derivative 18 with the carboxy-activated TG-resin 19. The ESI† provides detailed procedures for synthesizing nucleosides 17, 18, 3′-phosphoramidite cytidine derivative 21, and solid support 20.

### Solid-phase synthesis of ONs 1 and 3

**Scheme 2:**
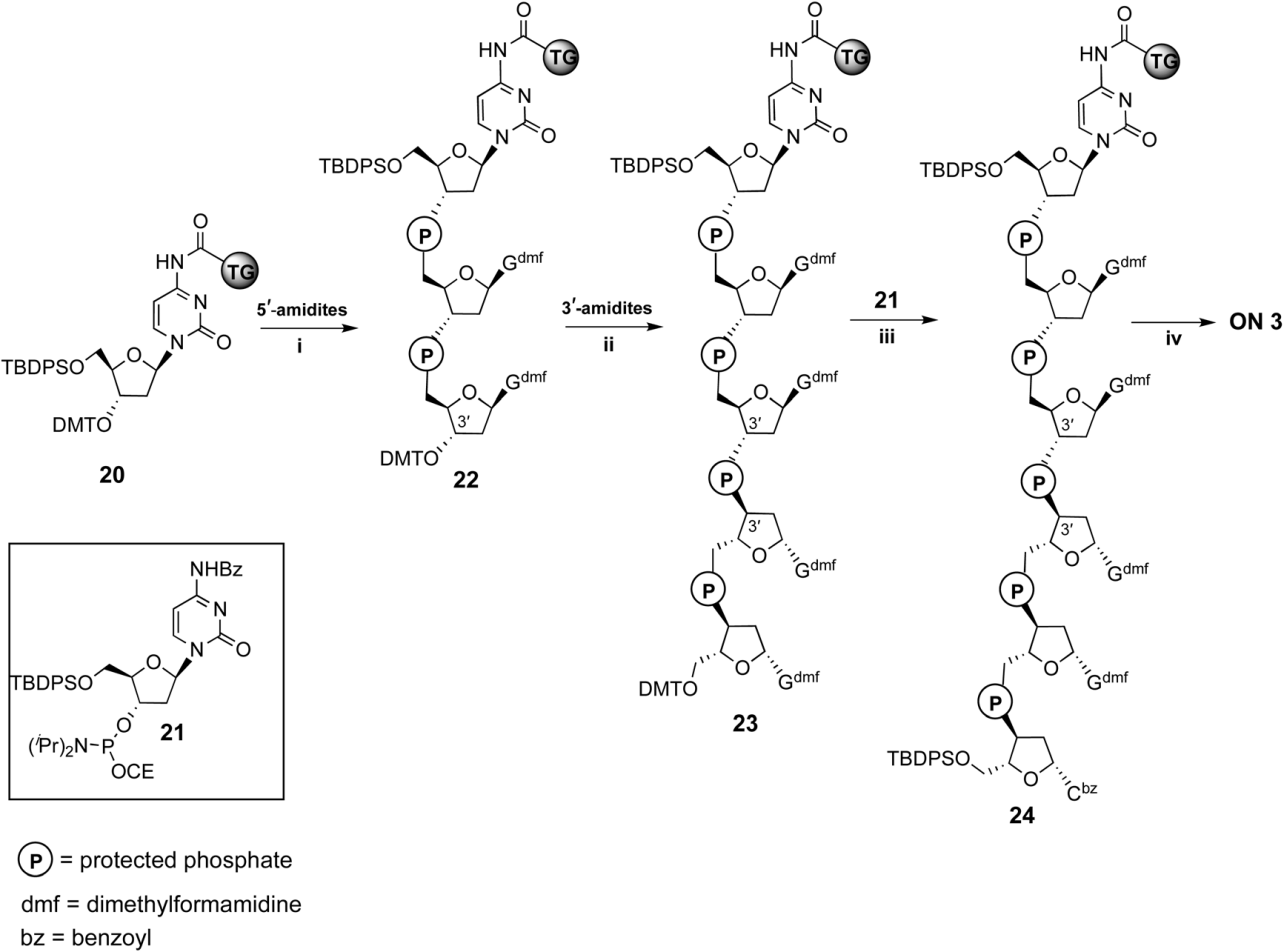
Synthesis of ON 3. Reagents and conditions: (i) two coupling cycles with *N*^2^-DMF-5′-O-P(OCE)[*N*(^i^Pr)_2_]-3′-O-DMT-dG, (ii) two coupling cycles with *N*^2^-DMF-5′-O-DMT-3′-O-P(OCE)[*N*(^i^Pr)_2_]-dG; (iii) last coupling cycle with phosphoramidite 21; (iv) detachment from solid support and deprotection with conc. NH_4_OH.

ONs 1 and 3 were synthesized using solid support 20 through the standard β-cyanoethyl phosphoramidite chemistry. The procedure for obtaining ON 3 is reported in Scheme 2 and follows the strategy detailed below. After DMT removal on support 20, two coupling cycles were performed with 5′-O-phosphoramidite-3′-O-DMT-dG, yielding intermediate solid support 22. After DMT removal, two coupling cycles were performed with the standard 3′-O-phosphoramidite-5′-O-DMT-dG, thus obtaining solid support 23, bearing an ON containing a 3′-3′ inversion of polarity. The last coupling cycle was carried out with the 3′-phosphoramidite 21, yielding support 24. The treatment of resin 24 with conc. NH_4_OH ensured the deprotection and the detachment of ON 3 from the solid support.

For the synthesis of shorter ON 1, two coupling cycles were performed on support 20, the first with the 5′-O-phosphoramidite-3′-O-DMT-dG and the second one with the 3′-O-phosphoramidite-5′-O-DMT-dG. Then, the last coupling cycle was performed with phosphoramidite 21. ON 1 was obtained after deprotection and detachment from the solid support, as described for ON 3. ONs 1 and 3 were purified by reverse-phase HPLC, and their structures confirmed by ESI MS data and ^1^H NMR spectroscopy (ESI). ONs 5 and 8 were prepared as previously described.^52^

### Annealing procedure to obtain G4s 2 and 4

Taking into consideration the greater G4-stabilizing ability of K^+^ compared to Na^+^ cations, and to assess the effect of different K^+^ concentrations on the formation of G4s 2 and 4, as well as their ordered lipophilic aggregates, ONs 1 and 3 (at a single-strand concentration of 1.0 mmol L^−1^) were dissolved in 100 mmol L^−1^ or 20 mmol L^−1^ K^+^ buffers (pH 7.0). The samples were annealed by heating at 90 °C for 5 min, followed by rapid cooling to 4 °C. After 24 h, they were analyzed by ^1^H NMR, circular dichroism (CD), and polyacrylamide gel electrophoresis (PAGE).

### Nuclear magnetic resonance (^1^H NMR) studies on ONs 1 and 3

The results obtained using this technique allowed us to demonstrate the formation of G4 complexes, assess their thermal stability, and evaluate the formation of supramolecular aggregates.

ONs 1 and 3 were investigated using water-suppressed ^1^H NMR experiments (H_2_O/D_2_O, 9 : 1) in comparison with related ONs 5 and 8 lacking TBDPS groups. These experiments could unambiguously confirm the formation of G4 species by detecting the exchange-protected H-1 imino proton signals of guanosines involved in G-tetrads (12.5–10.5 ppm region). Furthermore, the thermal stability of the G4 complexes was estimated by monitoring the intensity of imino signals at increasing temperatures (25, 45, 65, and 85 °C).

**Fig. 3:**
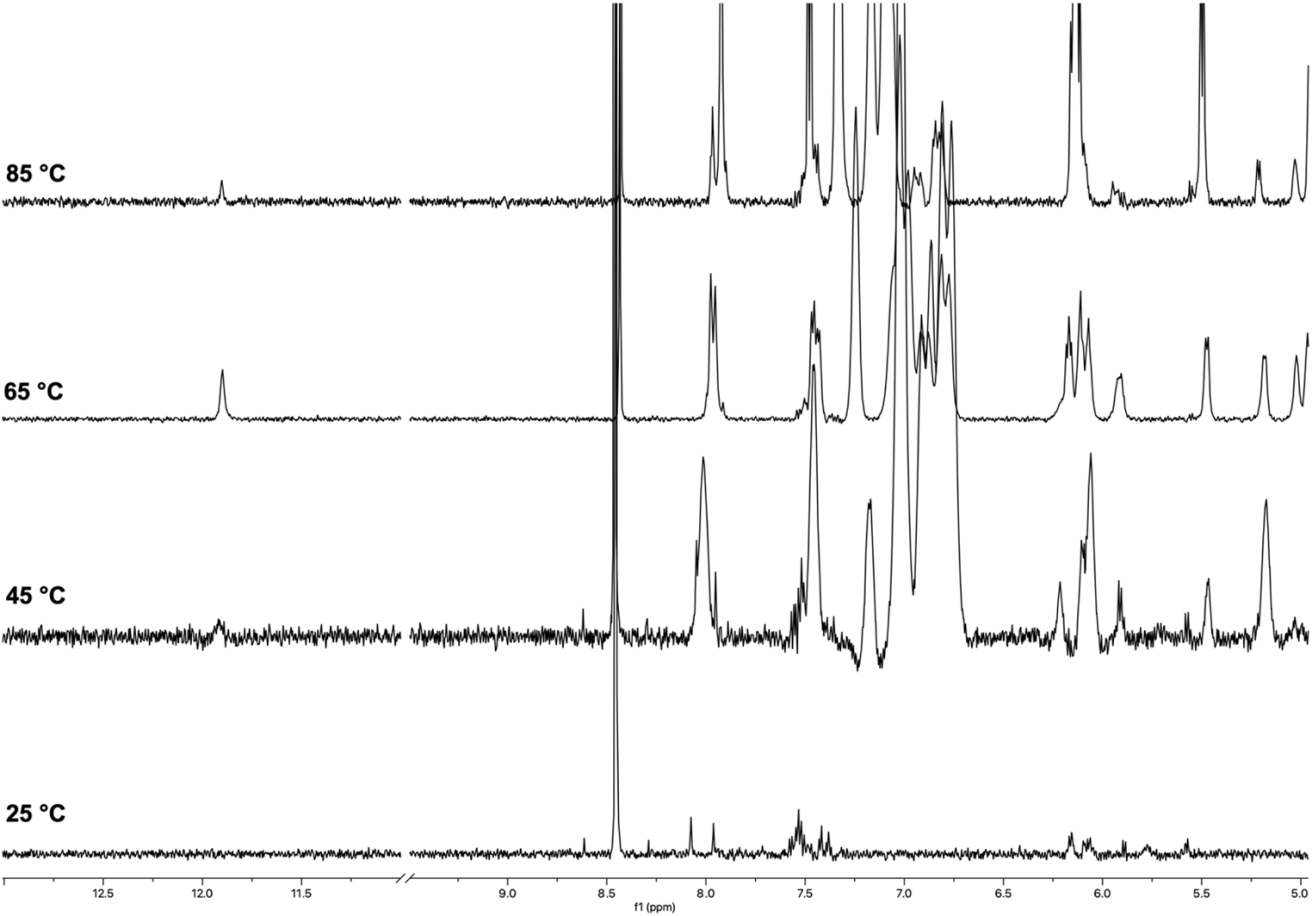
^1^H NMR spectra of ON 1 annealed in 100 mmol L^−1^ K^+^ buffer recorded at 25, 45, 65, and 85 °C.

The ^1^H NMR spectra of ON 1 annealed in 100 mmol L^−1^ K^+^ buffer at increasing temperatures are reported in Fig. 3. The spectrum recorded at 25 °C displayed signals with very low intensity in the entire 13.0–0.0 ppm region. Significant changes were observed in the spectrum as the temperature increased to 45 °C and 65 °C. At 45 °C, a broad imino proton signal appeared at 11.8 ppm, which became more intense and sharper at 65 °C. Simultaneously, intense and broad signals emerged in the aromatic and deoxyribose regions. This behavior could be attributed to the presence of high-molecular-weight G4 aggregates at lower temperatures (25–45 °C), which trapped the G4 units within a matrix, significantly reducing the detectability of protons in the ^1^H NMR spectrum. Most of these supramolecular aggregates dissociated in the 45–65 °C temperature range, releasing discrete G4 units. These units became detectable in the ^1^H NMR spectrum, as evidenced by the appearance of the single imino proton signal (11.8 ppm) attributable to the eight magnetically equivalent exchange-protected H-1 proton of G bases belonging to the two stacked symmetric G-tetrads in each G4 unit 2. An analysis of the aromatic and deoxyribose regions at 65 °C suggested the presence of an additional species, which we attributed to the single strand (SS) ON 1. Compared to the G4's NMR signals, these signals increased during the 45–65 °C transition and became the dominant ones in the spectrum at 85 °C. The very low intensity of the imino proton signal at 85 °C indicated that G4 structure 2 melted within the 70–80 °C temperature interval.

The ^1^H NMR spectra of ON 1 annealed in 20 mmol L^−1^ K^+^ buffer recorded at increasing temperatures (25–85 °C; Fig. S1†) exhibited a similar behavior, thus indicating the formation of G4 2 also at lower K^+^ concentration. However, we observed a slight decrease in the thermal stability of 2, as accounted by the absence of any imino proton signal at 85 °C. Despite the expected lower stability of the G4 in reduced K^+^ concentration, the G4 diagnostic imino proton signal was also detected at 25 °C. We attributed this difference to a reduced degree of G4 aggregation in the 20 mmol L^−1^ K^+^ buffer, which, along with the detection of the imino protons, determined the appearance in the spectrum of unquenched aromatic and deoxyribose signals.

Under the same experimental conditions, ON 5, having the same sequence as ON 1 but lacking the two TBDPS groups, failed to form corresponding G4 structure 9,^52^ thus unveiling the significant G-quadruplex stabilizing effect induced by the presence of the TBDPS groups at the 5′-end faces of ON 1. Notably, lipophilic G4 2, which exhibited remarkable thermal stability even at the lower K^+^ concentration (20 mmol L^−1^), represented the first reported tetramolecular G4 containing only two G-tetrads.

**Fig. 4:**
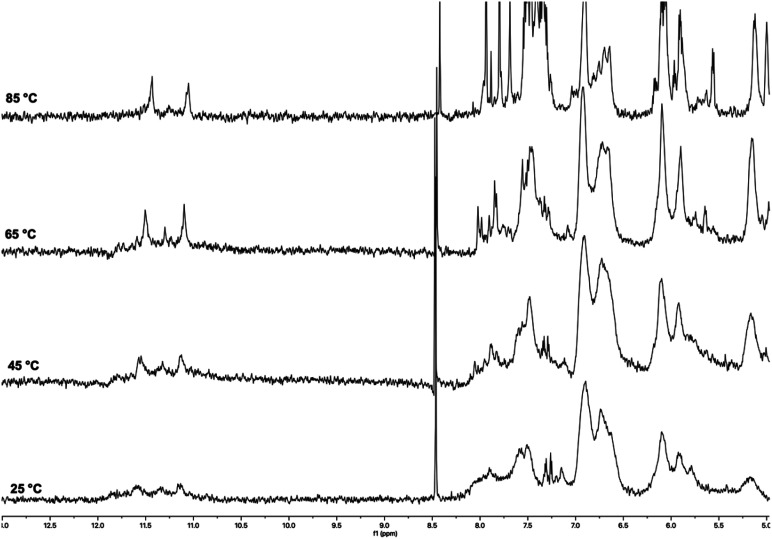
^1^H NMR spectra of ON 3 annealed in 100 mmol L^−1^ K^+^ buffer recorded at 25, 45, 65 and 85 °C.

The ^1^H NMR spectra of ON 3 annealed in 100 mmol L^−1^ K^+^ buffer recorded at increasing temperatures (25–85 °C) are shown in Fig. 4. At 25 °C, the spectrum displayed broad and poorly defined imino proton signals between 12.0 and 11.0 ppm and broad unresolved signals in the aromatic and deoxyribose regions. The number and shape of imino signals indicated that discrete G4 species (4) were present in solution together with high-molecular-weight G4 aggregates. However, the aggregation phenomena appeared to be reduced compared to those observed for G4 2 upon the same temperature and K^+^ concentration, as evidenced by only a partial quenching of the aromatic and deoxyribose signals at 25 °C. We observed significant changes in the ^1^H NMR spectra recorded in the 45–85 °C temperature range. At 45 °C, two distinct imino proton signals emerged at 11.2 and 11.3 ppm, which became sharper at 65 and 85 °C (Fig. 4). These signals, attributable to the two non-equivalent G-tetrads formed by the G2 and G3 bases of ON 3, confirmed the formation of symmetric G4 structure 4. Similarly, the aromatic and deoxyribose proton regions were populated by better-resolved signals at 85 °C. However, the complexity of these regions, likely due to the co-presence in the NMR tube of a substantial amount of single strand ON 3, prevented the assignment of NMR signals. We attributed the presence of more than two imino signals at 25 and 45 °C to the formation of G-wire assemblies, likely resulting from the multimerization of G4 building blocks (4) *via* 5′-5′ π–π stacking interactions between the G4 units.^53^ This multimeric arrangement, likely G-wire 14 (Fig. 2), was further stabilized by the formation of G(:C):G(:C):G(:C):G(:C) octads at both 5′-ends and dissociated within the temperature range 45–65 °C. The dissociation process resulted in the breakdown of the multimers, unmasking the discrete monomeric building block G4 4, characterized by only two imino proton signals.

At this point, it was reasonable to ask why G4 4 multimerized in stacked G-wires while G4 2 did not. This question was addressed in our previous work,^52^ where we demonstrated that 5′ C-G-ending G4s can multimerize through the formation of 5′-ending G(:C):G(:C):G(:C):G(:C) octads only when the G base within the 5′ C-G end is not involved into the 3′-3′ inverted phosphodiester bond. Based on these considerations, G4 4 was expected to multimerize into stacked G-wire structures, whereas G4 2 could not. This behavior also suggested that the four 5′-TBDPS groups did not hinder the 5′-5′ π–π stacking interactions among the G4 4 building blocks.

The ^1^H NMR spectra of ON 3 annealed in 20 mmol L^−1^ K^+^ buffer recorded at increasing temperatures are shown in Fig. S2.† The spectrum recorded at 25 °C showed two broad, well-resolved imino proton signals at 11.2 and 11.6 ppm, differently from the one acquired in 100 mmol L^−1^ K^+^ buffer at the same temperature in which no imino protons were detected. These signals, attributable to the G2 and G3 G-tetrads, confirmed the formation of G4 4 even at lower K^+^ concentration (20 mmol L^−1^). The two imino proton signals became more intense and sharper as the temperature rose to 85 °C, due to the release of discrete G4s 4 from the G4 aggregates under the temperature increase. The presence at 25 °C of aromatic and deoxyribose NMR signals indicated a reduced extent of G4 aggregation in the 20 mmol L^−1^ K^+^ buffer. The contemporaneous presence at 85 °C of sharp imino signals belonging to G4 4 and aromatic signals belonging to single-stranded 3 indicated that G4 4 melted in the 80–90 °C temperature interval. Notably, the corresponding G4 12, lacking the 5′-TBDPS groups, was significantly less stable than 4 in the 100 mmol L^−1^ K^+^ buffer, with a melting temperature in the 65–75 °C range, and remained almost stable up to 85 °C only in the presence of 1.0 mol L^−1^ K^+^.^52^

The results of ^1^H NMR investigations on G4s 2 and 4 in 100 and 20 mmol L^−1^ K^+^ buffers provided insights into the formation and structure of G4s formed by ON 1 and 3 and allowed us to propose hypotheses regarding the formation of G4 aggregates. In the case of TBDPS 5′-capped G4s, the formation and extension of G4 aggregates appeared to be more influenced by the structure of the G4 building block rather than by the K^+^ concentration, as previously observed for G4s lacking the lipophilic TBDPS groups. G4 2, with its shorter hydrophilic stem and a higher proportion of lipophilic shells, exhibited more extended G4 aggregation than G4 4, which had a longer hydrophilic stem and a lower proportion of lipophilic shells. Based on these observations, we attributed to the lipophilic interactions occurring between the terminal TBDPS shells surrounding the 5′-ends of the hydrophilic stem of G4s 2 and 4 a significant role in the aggregation phenomenon.

### Circular dichroism (CD) studies on annealed ONs 1 and 3

**Fig. 5:**
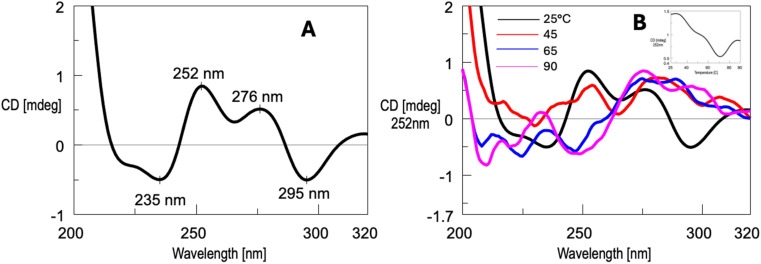
CD spectrum at 25 °C (A) and CD profile variation in the temperature range 25–90 °C (B) of ON 1 annealed in the 100 mmol L^−1^ K^+^ buffer. Insert: CD melting profile at *λ* = 252 nm.

Circular dichroism (CD) spectroscopy is a powerful and widely used technique for studying secondary structures of nucleic acids, including G4s. The method provides key information about such structures' formation, topology, and stability.^58,59^ The results obtained using this technique allowed us to demonstrate the formation of G4 complexes 2 and 4, assess their thermal stability, and suggest that the supramolecular aggregates are composed of G4 units. The CD spectrum of ON 1 annealed in the same 100 mmol L^−1^ K^+^ buffer used for the NMR analyses exhibited at 25 °C a profile characterized by two positive bands at 252 and 276 nm and two negative bands at 235 and 295 nm (Fig. 5A). The presence of well-defined bands suggested that ON 1 formed a structured complex, which was assigned to G4 2 considering the positions of the two positive bands which closely resembled those reported for G4s formed by d(5′-TG3′-3′GGT-5′)_4_ ^60^ and d(5′-CG3′-3′GGGC-5′)_4_,^52^ both containing a 3′-3′ inversion of polarity site. Notably, under the same conditions, ON 5, which lacked the 5′-TBDPS groups, displayed a CD profile typical of an unstructured random coil with bands of very low intensity. By increasing the temperature from 25 to 90 °C, we observed significant changes in the CD profile of ON 1 (Fig. 5B), which suggested the occurrence of structural changes. This finding appeared to contrast with the ^1^H NMR results, which indicated an increase of discrete G4 2 units in the 25–65 °C temperature range due to the gradual dissociation of the G4 aggregates, along with a corresponding increase in the amount of single-stranded species (SS). Such an apparent discrepancy could arise from the strong temperature dependence of the structural features of G4 aggregates. In contrast, ^1^H NMR experiments could not detect structural changes within G4 aggregates, offering information only on the amount of discrete G4 2 and SS 1 species. Based on these considerations, the CD_252_ melting profile (insert in Fig. 5B) could not be reliably used to calculate the melting temperature of G4 2. Instead, the melting temperature could be more accurately estimated following the ^1^H NMR profiles, which indicated in 100 mmol L^−1^ K^+^ buffer a value falling in the 70–80 °C range.

The CD spectrum at 25 °C of ON 1 annealed in the 20 mmol L^−1^ K^+^ buffer (Fig. S3A†) displayed a profile similar to that observed in the 100 mmol L^−1^ K^+^ buffer (Fig. 5A). This profile included two positive bands at 250 nm and 275 nm, along with negative bands at 231 nm and 298 nm, indicating the presence of a G4 structure even at this lower K^+^ concentration. This observation was consistent with the NMR data, which confirmed the presence of G4 2 under the same conditions (Fig. S1†). Upon increasing the temperature from 25 to 90 °C (Fig. S3B†), notable changes in the CD profile were observed, suggesting significant structural transitions. These changes, as corroborated by NMR analysis, could be attributed to the gradual melting of G4 2 aggregates with the concurrent increase of discrete units of G4 2 and SS ONs 1. The melting profile derived from the CD signal at 250 nm (inset, Fig. S3B†) revealed a well-defined melting curve, indicating a melting temperature for G4 2 in the range of 45–55 °C. This melting temperature was lower than that estimated by NMR under identical conditions, which could be attributed to the lower concentration of single-stranded ON 1 used in the CD experiments (0.01 mmol L^−1^) compared to the concentration employed in the NMR experiments (1 mmol L^−1^).

**Fig. 6:**
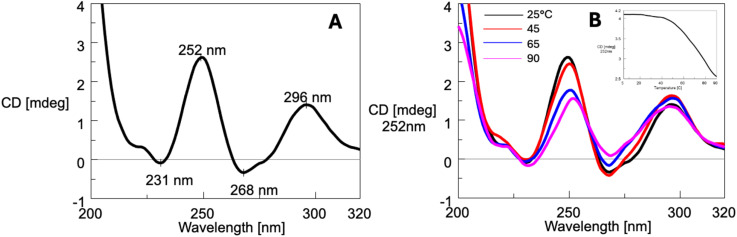
CD spectrum at 25 °C (A) and CD profile variation in the temperature range 25–90 °C (B) of ON 3 annealed in the 100 mmol L^−1^ K^+^ buffer. Insert: CD melting profile at *λ* = 252 nm.

The CD spectra of ON 3 at 25 °C (Fig. 6A), annealed in the 100 mmol L^−1^ K^+^ buffer, displayed two positive bands at 252 nm and 296 nm, along with two negative bands at 231 nm and 268 nm. The CD profile closely resembled that reported for the tetramolecular G4 formed by the oligonucleotide d(5′-TG-3′-3′-GGT-5′),^60^ as well as G4 12 (Fig. 2) formed by ON 8, d(5′-CGG-3′-3′-GGC-5′),^53^ both containing a 3′-3′ inversion of polarity site. These similarities supported the formation of G4 4 upon annealing of ON 3. An increase in the temperature from 5 to 90 °C (Fig. 6B) did not alter significantly the CD profile, indicating that G4 4 was highly stable and did not melt even at 90 °C. This latter finding was further supported by the incomplete sigmoidal profile of the CD_252_ melting profile (inset, Fig. 6B). All together, these observations were consistent with ^1^H NMR data, which confirmed the persistence of G4 4 up to 85 °C. Moreover, since NMR data suggested the presence of G4 aggregates at least up to 50 °C, it can be concluded that this supramolecular organization, unlike G4 2 aggregates, does not appreciably alter the CD profile.

The CD spectrum of ON 3 annealed in the 20 mmol L^−1^ K^+^ buffer (Fig. S4A†) exhibited a profile similar to that observed in the 100 mmol L^−1^ K^+^ buffer. Upon increasing the temperature from 25 to 90 °C (Fig. S4B†), the CD profiles remained unchanged, indicating that G4 4 was stable up to 90 °C. This observation aligned with NMR data, which also suggested the persistence of G4 4 under these conditions.

### Polyacrylamide gel electrophoresis (PAGE) studies

This study confirmed that G4s 2 and 4 form supramolecular aggregates and suggested that the extent of aggregation differs between the two structures.

**Fig. 7:**
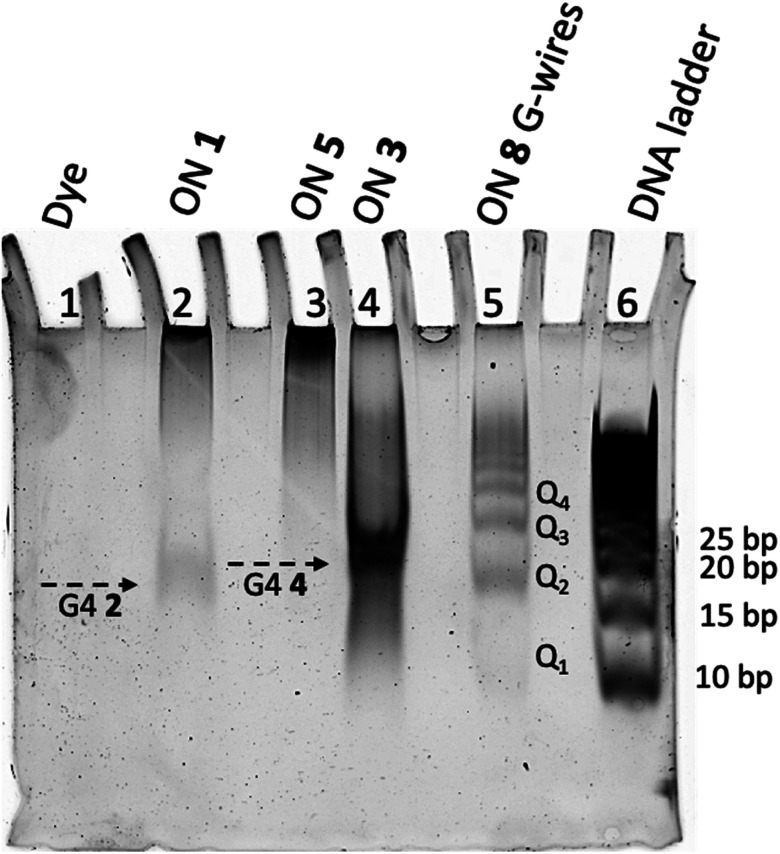
Non-denaturing PAGE analysis of ONs 1, 3, 5, and 8 after annealing in 100 mmol L^−1^ K^+^ buffer by SYBR Green visualization. With *Q*_2_–*Q*_4_ we indicated the G4 multimers formed by π–π stacking of two or more G4 building block 12 (*Q*_1_) formed by annealing the ON 8 (see Fig. 2).

TBDPS-modified ONs 1 and 3 and their unmodified counterparts, ONs 5 and 8, were analyzed post-annealing in the 100 mmol L^−1^ K^+^ buffer using PAGE electrophoresis (Fig. 7). As previously observed, ON 5 showed a smear pattern other than detectable bands (lane 3), which indicated that it does not form a G4 structure or any related multimers, as confirmed by NMR analysis.^52^ In contrast, ON 1, capped with the TBDPS groups, exhibited both a distinct band (marked by the arrow) and a less extended smearing (lane 2), which were consistent with the formation of G4 structure 2 and its associated aggregates. The mobility of the discrete band, corresponding to that of a 20 base pair-long ON (as estimated from the DNA ladder), appears unusually low for a single short G4 species. We attribute the reduced mobility of G4 2 to the presence of the eight bulky TBDPS groups attached to the 5′-faces of the G4 structure other than to the formation of a 5′-5′ stacked dimeric structure because such a complex would exhibit a different ^1^H NMR profile, characterized by two distinct imino protons due to the two nonequivalent G-tetrads. When ON 8 (lane 3) was analyzed under the same conditions, it migrated as a well-defined ladder of bands, with the fastest band corresponding to G4 12 depicted in Fig. 2. ON 3, having the same sequence as ON 8 and containing 5′-TBDPS groups, exhibited both smearing and an intense band (marked by the arrow in lane 4), which we attributed to the formation of G4 structure 4. The mobility of G4 4 band was slightly lower than that of G4 2, consistent with the longer sequence of ON 3. Additionally, the reduced mobility of G4 4 (corresponding to that of a 20–25 base pairs-long ON) can be attributed to the presence of the eight bulky TBDPS groups attached at the 5′ faces of the G4 structure, which also caused the formation of the lipophilic G4 4 aggregates responsible for the smearing. However, based on this analysis we cannot rule out the possibility that G4 4 forms stacked multimers similar to those formed by G4 12. In this scenario, the well-defined migration of *Q*_*n*_ multimer bands may be disrupted by the presence of the lipophilic aggregates.

### Morphological characterization of lipophilic G4 aggregates by SEM, DLS, and AFM analyses

To get insight into the morphology of G4 aggregates formed by G4 2, we used SEM, DLS, and AFM techniques, which have already been successfully used to explore the morphology of several types of G4 architectures.^46,61,62^ Fig. S12A–C† presents representative SEM images of the dried G4 2 sample. The SEM analysis reveals the presence of submicrometric spherical aggregates, with diameters ranging from approximately 40–50 nm up to 700–800 nm. These structures suggest the coexistence of multiple aggregation states. As shown in Fig. S12A,† concave aggregates are observed in less densely populated sample regions, typically located in the central area of the drop halo. This distribution pattern suggests that these features may result from material deposition occurring after the application of mechanical stress, likely due to the drying process or exposure to SEM vacuum conditions. The morphology of these concave structures is consistent with hollow spheres, such as micelles, which may rupture under low vacuum.

The SEM data were secured by DLS measurements, which confirmed the polydisperse nature of the sample, revealing the presence of particles with an average hydrodynamic diameter of 600 ± 100 nm and a polydispersity index (PDI) of 0.68 (Fig. S12D†).

To enable the imaging and measurement of the smallest aggregate particles, samples were deposited onto an ultra-flat substrate, specifically freshly cleaved muscovite mica. Following deposition, the substrate was rinsed to remove residual salt crystals, and the samples were subsequently analyzed using atomic force microscopy. AFM represents one of the most powerful techniques for label-free nanoscale characterization of aptamer-based nanostructures, as it facilitates the confirmation of predicted dimensions, shapes, and other physicochemical properties.^54^

In this study, AFM was employed to characterize nanostructures spontaneously adsorbed onto mica surfaces and investigate the potential formation of supramolecular assemblies. Muscovite mica was selected as the substrate for AFM imaging due to its exceptional flatness, with a root-mean-square (RMS) roughness of less than 0.5 nm over a 1000 × 1000 nm^2^ area. In addition, mica cleavage ensures a clean, hydrophilic surface that exhibits good wettability and favors the adsorption of negatively charged molecules.^54,63^

**Fig. 8:**
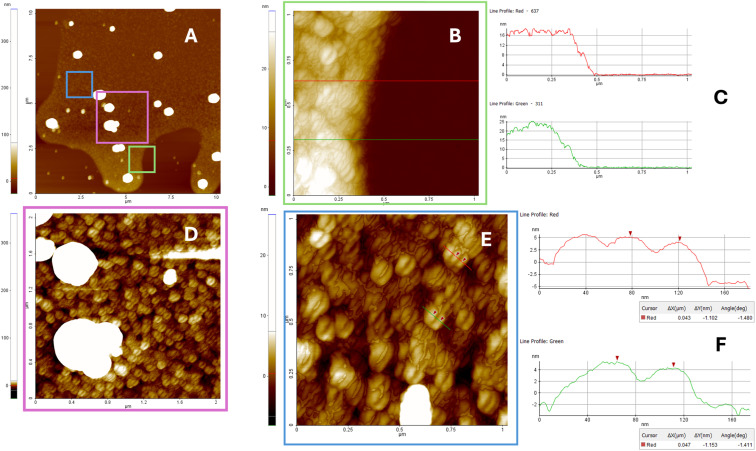
AFM analysis of G4 2 aggregates (Panels A–D) was conducted on samples deposited on a mica substrate and rinsed after three minutes of interaction. Panel A shows particles distributed over a broad area at the periphery of the stain formed during deposition. Panels B, D, and E present magnified views of selected regions from Panel A. Panels C and F display height profiles measured along lines indicated in Panels B and E, respectively. The smallest observed feature measures approximately 40 nm, in agreement with the dimensions observed in SEM images.

Fig. 8 presents the AFM characterization of G4 2 aggregates on mica after three minutes of interaction. Aggregates with coffee bean-like morphology and diameters of approximately 40 nm were observed, along with larger structures measuring around 300 nm. These findings suggest that the selected deposition method on the hydrophilic mica surface promotes the preferential adsorption of smaller micelles over larger aggregates. As with the SEM imaging, AFM analysis was performed on particles located at the periphery of the stain formed during deposition, initially over a broad area, followed by a more detailed investigation of the smallest regions where nanoaggregates were present. No evidence of collapsed micelles or concave structures was detected in the AFM analysis. This observation is consistent with expectations, as measurements were conducted under ambient conditions, specifically in air at room temperature and atmospheric pressure.

## Conclusions

In this work, we continued our investigation into G-rich oligonucleotides (ONs) containing a 3′-3′ inversion of polarity and their ability to form G4 structures and ordered multimeric G-wire assemblies. Specifically, we examined the effects of introducing two TBDPS groups at the 5′-ends of two oligonucleotides, 1 and 3, having sequences d(TBDPS-5′-CG_*n*_-3′-3′-G_*n*_C-5′-TBDPS) (*n* = 1 or 2). The presence of the two TBDPS groups imparted a lipophilic feature to ONs 1 and 3 and did not hinder the formation of G4 2 and 4. The four TBDPS groups attached to the 5′ ends of each G4 strand significantly enhance the stability of the G-quadruplexes, likely through van der Waals interactions. This stabilizing effect lowers the potassium ion concentration required for G4 formation and imparts amphiphilic properties to the resulting structures. G4 2 represents the first reported tetramolecular G4 containing only two G-tetrads. Remarkably, this lipophilic G4 exhibits high stability even at low K^+^ concentration (20 mmol L^−1^). In contrast, the corresponding non-lipophilic G4 9 fails to form, even at elevated K^+^ concentrations (up to 1 mmol L^−1^). A similar trend was observed for G4 4, which was clearly detectable at 20 mmol L^−1^ K^+^ and demonstrated greater stability than its non-TBDPS-labeled analogue, G4 12.

In general, lipophilic G4s could interact with lipid molecules, paving the way for developing new amphiphilic DNA-based products, which could be exploited for applications in drug delivery or membrane targeting. Furthermore, the here-reported G4s exhibited a strong tendency to form supramolecular aggregates, which we attributed to lipophilic interactions between the terminal TBDPS shells surrounding the 5′-ends of the hydrophilic G4 stems. The stability and extent of the G4 aggregates appeared to be significantly influenced by the structural features of the G4s, particularly by the ratio between the size of the lipophilic TBDPS shells and the hydrophilic G4 stem. Indeed, G4 2, with a very short hydrophilic stem composed of only two G-tetrads, formed more extensive lipophilic aggregates. Although the structural features of these G4 aggregates have not been explored in detail in this study, we hypothesize that they could exhibit amphiphilic properties and potentially form bilayer structures with well-defined lipophilic and hydrophilic layers. Notably, the strong stabilization of these G4s and their ability to form lipophilic aggregates, whose stability can be modulated by temperature and cations concentration, highlight their potential for the development of novel DNA-based nanomaterials and drug delivery systems. Moreover, this new class of stable G4s and their related lipophilic aggregates warrants further investigation regarding their ability to interact with small molecules, such as drugs, as well as with protein enzymes, potentially impacting various cellular processes.

Regarding the synthetic procedure proposed here to obtain ONs 1 and 3, we revisited and optimized our previous protocol, which employed solid supports loaded with deoxycytidine *via* its *N*^4^-amino exocyclic group. This loading strategy leaves the 5′ and 3′ positions of the deoxyribose available for further modifications or functionalization with lipophilic, hydrophilic, or labeling groups. The solid-phase synthetic strategy enables the production of longer bis-labelled oligonucleotides (ONs) at both termini (3′ and 5′), incorporating a variety of functional groups beyond TBDPS. It also permits the incorporation of one or more inversions of polarity within the ON strand, allowing for the synthesis of both symmetric and asymmetric ONs, with or without bis-labelling. Importantly, this methodology—based on standard phosphoramidite chemistry—is fully compatible with automated DNA synthesizers, making it well suited for large-scale production.

## Data availability

The data supporting this article have been included as part of the (ESI).†

## Author contributions

Maria Marzano: investigation, methodology. Maria Grazia Nolli: investigation, software. Stefano D'Errico: conceptualization, formal analysis, methodology, writing original draft, supervision. Andrea Patrizia Falanga: data curation. Principia Dardano: investigation, methodology. Luca De Stefano: data curation, formal analysis. Monica Terracciano: formal analysis. Gennaro Piccialli: data curation, funding acquisition, writing original draft. Nicola Borbone: conceptualization, data curation, funding acquisition writing – review & editing. Giorgia Oliviero: formal analysis, methodology, writing – review & editing, supervision.

## Conflicts of interest

There are no conflicts to declare.

## Supplementary Material

RA-015-D5RA01033K-s001
