# Supplementary material for: Enhancing G-quadruplex-based DNA nanotechnology: new lipophilic DNA G-quadruplexes with TBDPS modifications
Source: RSC Adv. 2025 May 29;15(23):17933–45. doi: 10.1039/d5ra01033k (PMC12120934; doi:10.1039/d5ra01033k)
Supplement: RA-015-D5RA01033K-s001 [file RA-015-D5RA01033K-s001.pdf]

Supplementary Information (SI) for RSC Advances.
This journal is © The Royal Society of Chemistry 2025

SUPPORTING INFORMATION

Enhancing G-Quadruplex-Based DNA Nanotechnology: New Lipophilic DNA G-quadruplexes with TBDPS Modifications

Maria Marzano[a], Maria Grazia Nolli[a], Stefano D'Errico[a,*], Andrea Patrizia Falanga[a], Monica Terracciano[a], Principia Dardano[b], Luca De Stefano[b], Gennaro Piccialli[a,c], Nicola Borbone[a,c,*] and Giorgia Oliviero[c,d]

[a]Dipartimento di Farmacia, Università degli Studi di Napoli Federico II, Via Domenico Montesano 49, 80131 Napoli, Italy

[b]Institute of Applied Sciences and Intelligent Systems "Eduardo Caianiello", Unit of Naples, National Research Council, Naples, 80131, Italy

[c]ISBE-IT, Università degli Studi di Napoli Federico II, 80138 Napoli, Italy

[d]Dipartimento di Medicina Molecolare e Biotecnologie Mediche, Università degli Studi di Napoli Federico II, Via Sergio Pansini 5, 80131 Napoli, Italy

* Corresponding authors: Stefano D'Errico (stefano.derrico@unina.it) and Nicola Borbone (nicola.borbone@unina.it)

General

All reagents and solvents were commercially available and used without further purification. Flash chromatography was performed on a Biotage Selekt Enkel instrument (Biotage Sweden AB, Uppsala, Sweden). TLC analyses were performed on 0.2 mm thick F_{254} silica gel plates (Merck, Darmstad, Germany). TLC spots detected under UV light (254 nm). ONs were produced through the Expedite 8909 DNA automated synthesizer (PerSeptive Biosystems, Cumming, GA, USA). Support **20** was prepared by using the carboxy activated TentaGel resin **19** (Rapp Polymere GmbH, Tuebingen, Germany). ONs **1** and **3** were purified by HPLC on a Jasco PU-4180 Plus instrument equipped with a Jasco UV-4075 Plus UV detector (Jasco Europe s.r.l., Milan, Italy), using a Purosphere Star 4.8 ×150 mm C-18 reverse-phase column with particle size 5 μm (Merck) eluted with a linear gradient of CH_3CN in 0.1 M

triethylammonium bicarbonate (TEAB) buffer (from 0 to 100 % in 45 min, flow 2.0 mL/min). UV spectra were acquired on a Jasco V-730 spectrophotometer. CD spectra were recorded on a Jasco 1500 spectropolarimeter equipped with a Jasco PTC-348-WI temperature controller. ^{1}H, ^{13}C and ^{1}H-decoupled ^{31}P NMR spectra were acquired on Bruker Avance Neo 400, 600 and 700 MHz spectrometers equipped with a broadband inverse probe with z-field gradients and an HCN triple resonance CryoProbe (Bruker-Biospin, Billerica, MA, USA) using $CDCl_3$, DMSO-d_6, acetone-d_6 and CD_3OD as solvents. NMR chemical shifts are reported in parts per million (δ) relative to the residual solvent signals: $(CH_2D)CD_3SO$ 2.54, $(CH_2D)CD_3CO$ 2.09, CD_2HOD 3.31 for ^{1}H-NMR and DMSO-d_6 40.4, acetone-d_6 30.6 and CD_3OH 49.0 for ^{13}C-NMR. The ^{1}H NMR chemical shifts were assigned through 2D NMR experiments. ^{1}H-decoupled ^{31}P NMR spectra were acquired using 85% H_3PO_4 as an external standard (0 ppm). The NMR spectra were processed using the MestReNova 14.3.1 software suite (Mestrelab Research, Santiago de Compostela, Spain). ESI MS spectra were acquired on a 4000 QTRAP (AB Sciex, Framingham, MA, USA) and an LTQ-XL (ThermoScientific, Waltham, MA, USA) mass spectrometers.

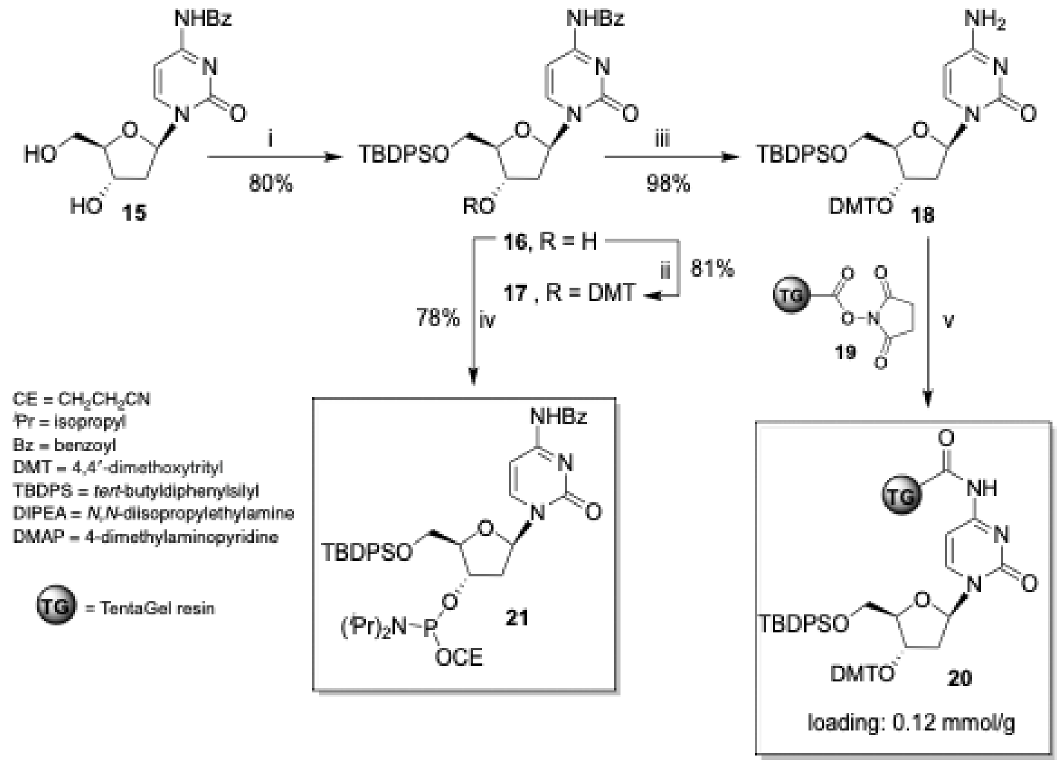

Scheme 1. Reagents and Conditions: i) TBDPSCl, imidazole, pyridine, r.t., 4 h; ii) DMTCl, DMAP, pyridine, 40 °C, 16 h; iii) NH_3 in CH_3OH, r.t., 2 h; iv) (^{i}Pr)$_2$NP(Cl)OCE, DIPEA, CH_2Cl_2, r.t., 2 h; v) **19**, CH_2Cl_2, r.t., 16 h. TG = TentaGel resin.

N^4-Bz-5'-O-TBDPS-3'-O-DMT-2'-dC ***17***

To a stirred solution of compound **16** (600 mg, 1.1 mmol, Scheme 1) in anhydrous pyridine (6.0 mL), DMAP (cat.) and DMTCl (720 mg, 2.2 mmol) were added. After 16 h at 40 °C (TLC monitoring: petroleum ether/AcOEt, 3:7 with 0.5% triethylamine), the reaction mixture was cooled to r.t. and the solvent was removed under reduced pressure. The crude mixture was purified by silica gel flash chromatography (from 0 to 50% AcOEt in petroleum ether with 0.5% v/v triethylamine in 30 min, 80 mL/min, Biotage Sfar 25 g column), giving pure **17**. Colorless foam (81% yield). ^{1}H NMR (400 MHz, DMSO-d_6) δ 11.31 (s, 1H, NH), 8.18 (d, *J* = 7.5 Hz, 1H, 6-H), 8.09–7.98 (m, 2H, *ortho*-Bz), 7.70–7.25 (complex signal, 20H, arom. and 5-H), 7.21 (d, 6.87, *J* = 8,8 Hz, 4H, *meta*-DMT), 6.87 (t, *J* = 9.0 Hz, 4H, *ortho*-DMT), 6.24 (dd, *J* = 8.0, 5.8 Hz, 1H, 1'-H), 4.17 (d, *J* = 5.8 Hz, 1H, 3'-H), 3.80 (bs, 1H, 4′-H), 3.75 (s, 3H, OCH_3), 3.74 (s, 3H, OCH_3), 3.71 (m, 1H, partially covered by residual solvent signal, 5'-H_a), 3.33 (dd, *J* = 11.9, 3.8 Hz, 1H, partially covered by residual solvent signal 5'-H_b), 2.25 (dd, *J* = 13.5, 5.8 Hz, 1H,

2'-H_a), 1.84–1,73 (m, 1H, 2'-H_b), 0.93 (s, 9H, ^{t}Bu). ^{13}C NMR (101 MHz, DMSO-d_6) δ 168.2, 163.9, 159.1, 155.1, 145.9, 144.9, 136.8, 136.6, 136.0, 135.9, 134.0, 133.6, 133.1, 133.0, 130.9, 130.7, 130.6, 129.3, 128.9, 128.8, 128.7, 128.6, 127.8, 114.2, 96.8, 87.9, 87.6, 87.3, 75.4, 65.8, 55.9, 55.8, 41.5, 27.4, 19.5. ESI MS *m/z* 872, ([M + H]$^+$ calcd. for $C_{53}H_{54}N_3O_7Si$ 872).

N^4-Bz-5'-O-TBDPS-3'-O-P(OCE)[N(^{i}Pr)$_2$]-2'-dC ***21***

To a stirred solution of compound **16** (500 mg, 0.88 mmol) in anhydrous dichloromethane (5.0 mL), DIPEA (0.58 mL, 3.3 mmol) and then (^{i}Pr)$_2$NP(Cl)OCE (0.30 mL, 1.4 mmol) were added. After 2 h at r.t. (TLC monitoring: petroleum ether/AcOEt, 3:7 with 0.5% triethylamine), the reaction mixture was diluted with a 5% $NaHCO_3$ aqueous solution (30 mL) and extracted with AcOEt (2 × 30 mL) The organic layers were collected, washed with brine (30 mL), dried over anhydrous Na_2SO_4 and filtered. The solvents were removed under reduced pressure and the crude mixture was purified by silica gel flash chromatography (from 0 to 50% AcOEt in petroleum ether with 0.5% v/v triethylamine in 30 min, 80 mL/min, Biotage Sfar 25 g column), giving pure **21** as a 1:1 mixture of diastereomers. Colorless foam (78% yield). ^{1}H NMR (400 MHz, Acetone-d_6) δ 8.37–8.26 (two overlapped d, *J* = 7.7 and 7.2 Hz, 2H, 2 × 6-H), 8.29 (d, *J* = 7.4 Hz, 4H, 2 × *ortho*-Bz), 7.86–7.45 (complex signal, 28H, 2 × arom. and 2 × 5-H), 6.35–6.29 (complex signal, 2H, 2 × 1'-H), 4.82–4.73 (complex signal, 2H, 2 × 4'-H), 4.33–4.28 (m, 1H, 3'-H), 4.27–4.30 (m, 1H, 3'-H), 4.11 (td, *J* = 11.8, 3.4 Hz, 2H, CH_2OP), 4.05–3.65 (complex signal, 10H, CH_2OP, 2 × 5'-$H_{a,b}$, 4 × CHN), 2.83–2.69 (complex signal, 6H, 2 × CH_2CN and 2 × 2'-H_a), 2.44–2.33 (complex signal, 2H, 2 × 2'-H_b), 1.27–2.33 (two overlapped d, *J* = 6.7 and 6.6 Hz, 18H, 3 × CH(CH_3)$_2$), 1.21 (d, *J* = 6.8 Hz, 6H, CH(CH_3)$_2$), 1.15 (s, 9H, ^{t}Bu), 1.14 (s, 9H, ^{t}Bu). ^{31}P NMR (162 MHz, Acetone-d_6) δ 148.5 (s), 148.3 (s). ^{13}C NMR (101 MHz, Acetone) δ 168.6, 164.3, 155.6, 145.7, 137.3, 137.2, 137.1, 137.0, 135.4, 134.6, 134.4, 134.3, 131.8, 131.7, 131.6, 130.2, 129.9, 129.6, 119.7, 119.6, 97.7, 88.5, 88.5, 88.3, 88.3, 88.2, 74.6 (d, *J* = 16.8 Hz), 74.2 (d, *J* = 17.4 Hz), 65.1, 60.4 (d, *J* = 8.7 Hz), 60.2 (d, *J* = 9.4 Hz), 55.7, 44.8 (d, *J* = 5.8 Hz), 44.7 (d, *J* = 5.9 Hz), 44.6, 42.2, 42.1, 42.0, 31.3, 31.2, 31.0, 30.8, 30.6, 30.4, 30.2, 30.0, 28.2, 28.1, 25.6, 21.5 (d, *J* = 4.3 Hz), 21.4 (d, *J* = 4.1 Hz), 20.6. ESI MS *m/z* 770, ([M + H]$^+$ calcd. for $C_{41}H_{53}N_5O_6PSi$ 770).

*5'-O-TBDPS-3'-O-DMT-2'-dC **18***

Compound **17** (400 mg, 0.46 mmol) was dissolved in 4.0 mL of a 7 M NH_3 methanolic solution and stirred at 55 °C for 3 h (TLC monitoring: AcOEt/MeOH, 9:1 with 0.5% triethylamine). After cooling the reaction mixture to r.t., the solvents were removed under reduced pressure and the crude mixture was purified by silica gel flash chromatography (from 0 to 2% CH_3OH in AcOEt with 0.5% v/v triethylamine in 30 min, 80 mL/min, Biotage Sfar 25 g column), giving pure **18**. Colorless glass (98% yield). ^{1}H NMR (400 MHz, CD_3OD) δ 7.85 (d, *J* = 7.5 Hz, 1H, 6-H), 7.58–7.13 (complex signal, 19H, arom.), 6.80–6.70 (complex signal, 4H, *ortho*-DMT), 6.35 (dd, *J* = 8.7, 5.4 Hz, 1H, 1'-H), 5.69 (d, *J* = 7.5 Hz, 1H, 5-H), 4.32–4.27 (m, 1H, 3'-H), 3.71 (s, 3H, OCH_3), 3.70–3.68 (complex signal, 4H, OCH_3 and 5'-H_a), 3.30–3.25 (m, 1H, partially covered by residual solvent signal, 5'-H_b), 2.25–2.17 (m, 1H, 2'-H_a), 1.85–1.74 (m, 1H, 2'-H_b), 0.94 (s, 9H, ^{t}Bu). ^{13}C NMR (101 MHz, CD_3OD) δ 167.5, 160.3, 160.2, 158.1, 146.7, 142.09, 137.7, 137.4, 136.7, 136.6, 133.9, 133.7, 131.5, 131.3, 131.2, 131.1, 129.4, 129.1, 129.0, 128.9, 128.0, 114.3, 114.2, 95.9, 88.6, 88.0, 87.9, 76.4, 65.5, 42.2, 27.5, 20.9, 20.0. ESI MS *m/z* 768, ($[M + H]^+$ calcd. for $C_{46}H_{50}N_3O_6Si$ 768).

Preparation of solid support **20**

Resin **19** (100 mg, loading: 0.22 mmol/g) was swelled in dry CH_2Cl_2 (1.0 mL) in a glass column (10 mm diameter, 100 mm length) with fused-in sintered glass disc PO (bore of plug 2.5 mm). A solution of compound **18** (84 mg, 0.11 mmol) in dry CH_2Cl_2 (1.0 mL) was added to the resin. The mixture was gently shaken at r.t. for 16 h. The obtained solid support **20** was drained, washed with CH_2Cl_2 (3 × 5 mL), CH_3OH (3 × 5 mL) and dried under reduced pressure. The loading of solid support **20** (0.12 mmol/g) was calculated by quantifying spectrophotometrically the amount of the DMT cation released (3% trichloroacetic acid in CH_2Cl_2) from a weighted amount of the resin-bonded nucleoside (λ_{max} = 502 nm, ε = 71,700 M^{-1} cm^{-1}).

*DNA Synthesis and purification of ONs **1** and **3***

ONs **1** and **3** were synthesized on the solid support **20** following the standard β-cyanoethyl phosphoramidite chemistry at 15 μmol scale by using 5'-phosphoramidite and standard 3'-phosphoramidite nucleotides.

For ON **3**, 50 mg of support **20** (0.006 mmol) in the automated DNA synthesizer, after DMT removal two coupling cycles were performed with 5'-phosphoramidite-3'-DMT-dG, (50 mg/mL CH_3CN solution). After DMT removal, two coupling cycles were performed by using 3'-phosphoramidite-5'-DMT-dG (50 mg/mL CH_3CN solution). The last coupling cycle was carried out with the 3'-phosphoramidite **21** (50 mg/mL CH_3CN solution). After washings, the dried resin was treated with 2 mL of conc. NH_4OH (12 h, 50 °C). The resulting solution and washings were dried and purified by HPLC (see General). The same procedure was used for the synthesis of ON **1**. In the latter case, only one coupling step with 5'-phosphoramidite-3'-DMT-dG and 3'-phosphoramidite-5'-DMT-dG were performed, respectively.

*d(TBDPSO-5'-CG-3'-3'-GC-5'-OTBDPS) (ON **1**)*

^{1}H NMR (600 MHz, D_2O, 85 °C) δ 7.87 (s, 2H, 2 × 8-H), 7.53 (d, *J* = 7.5 Hz, 2H, 2 × 6-H), 7.51 (d, *J* = 7.4 Hz, 8H, 2 × arom.), 7.43–7.36 (m, 4H, 2 × arom.), 7.36–7.27 (m, 8H, 2 × arom.), 6.18–6.13 (m, 2H, 2 × 1'-H), 6.10 (dd, *J* = 8.7, 5.8 Hz, 2H, 2 × 1'-H), 5.54 (d, *J* = 7.5 Hz, 2H, 2 × 5-H), 4.94–4.88 (m, 2H, 2 × 3'-H), 4.83–4.78 (m, 2H, 2 × 3'-H), 4.35–4.31 (m, 2H, 2 × 4'-H), 4.20–4.00 (complex signal, 6H, 2 × 4'-H and 2 × 5'-$H_{a,b}$, partially covered by residual solvent signal), 3.69 (apparent d, *J* = 2.9 Hz, 4H, 2 × CH_2OTBDPS), 2.70–2.62 (m, 2H, 2 × 2'-H), 2.59 (ddd, *J* = 14.2, 6.4, 3.1 Hz, 2H, 2 × 2'-H), 2.54 (dd, *J* = 13.9, 5.8 Hz, 2H, 2 × 2'-H), 2.20–2.13 (m, 2H, 2 × 2'-H), 0.87 (s, 18H, 2 × ^{t}Bu). ESI MS *m/z* calcd. for: $[M - H]^-$ 1649, found 1649; $[M - 2H]^{2-}$ 821, found 824.

*d(TBDPSO-5'-CGG-3'-3'-GGC-5'-OTBDPS) (ON **3**)*

^{1}H NMR (700 MHz, DMSO-d_6/D_2O, 9:1, 45 °C) δ 7.97 (s, 2H, 2 × 8-H), 7.88 (s, 2H, 2 × 8-H), 7.59 (d, *J* = 7.5 Hz, 2H, 2 × 6-H), 7.59–7.54 (m, 8H, 2 × arom.), 7.43–7.33 (m, 12H, 2 × arom.), 6.17 (apparent t, *J* = 6.9 Hz, 2H, 2 × 1'-H), 6.14 (apparent t, *J* = 7.5 Hz, 1H, 2 × 1'-H), 6.11 (apparent t, *J* = 7.4 Hz, 1H, 2 × 1'-H), 5.56 (d, *J* = 7.4 Hz, 2H, 2 × 5-H), 4.85 (bs, 2H, 2 × 3'-H),

4.81 (bs, 2H, 2 × 3'-H), 4.72 (bs, 2H, 2 × 3'-H). ESI MS *m/z* calcd. for: $[M - 2H]^{2-}$ 1154, found 1154; $[M - 3H]^{3-}$ 769, found 769.

Annealing procedure

The ON concentrations were measured spectrophotometrically in water at λ = 260 nm (90 ∘C), using the molar extinction coefficients ε = 35,120 M^{-1} cm^{-1} for ON **1**, and ε = 55,320 M^{-1} cm^{-1} for ON **3**, calculated using the Sigma-Aldrich OligoEvaluator™ web tool (www.oligoevaluator.com) and adding the phenyl contribute on the TBDPS groups (ε = 230 M^{-1} cm^{-1} for each phenyl group). The ONs were dissolved in 100 mM K^+ buffer (90 mM KCl and 10 mM KH_2PO_4) and 20 mM K^+ buffer (18 mM KCl and 2 mM KH_2PO_4), pH 7.0, at 1.0 mM single strand (SS) concentration. The ON samples were annealed by heating at 90 °C for 10 min and then quickly cooled to 4 °C. Thereafter, the ONs were stored at 4 °C before analyses.

*^{1}H NMR of the annealed ONs **1** and **3***

^{1}H NMR spectra were acquired at 16,384 data points with a recycle delay of 1.0 s at 10, 25, 45, 65, and 85 °C and the spectra were apodized with a shifted sine bell squared window function. Water suppression was achieved by including a double pulsed-field gradient spin-echo (DPFGSE) module in the pulse sequence prior to acquisition. NMR samples were prepared at the 1.0 mM single strand concentration in 200 μL H_2O/D_2O, 9:1, in 20 and 100 mM K^+ phosphate buffers.

Non-denaturing polyacrylamide gel electrophoresis (PAGE)

Native gel electrophoresis analyses were performed on 15 % polyacrylamide gels containing TBE 1× (8.9 mM Trizma® base, 8.9 mM borate, 0.2 mM EDTA) and 30 mM KCl, pH 7.0, at 120 V for 1.5 h at 25 °C. The annealed ONs (1 mM SS) were diluted at 0.1 mM SS loading concentration just before PAGE runs. Glycerol was added (10 % v/v final) to facilitate sample loading in the wells. The gels were treated with SYBR Green® (Bio-Rad Laboratories S.r.l., Segrate, Italy) and visualized by the Bio-Rad Gel Doc™ GO apparatus.

Scanning electron microscopy (SEM)

In order to image organic compounds or cells using Scanning Electron Microscopy (SEM), it is common practice to deposit a conductive layer onto the surface of the specimen. However, coating organic samples often results in the loss of nanometric features. To preserve such fine details while ensuring sufficient contrast for imaging organic nanosystems, the authors in [pia1] employed an alternative approach by depositing a diluted sample onto a conductive substrate. Specifically, an aqueous solution of G4 2TBDPS was deposited by drop casting (3 µL) onto a gold surface and allowed to dry under a fume hood. Imaging was performed using a Field Emission Scanning Electron Microscope (Gemini FESEM, Carl Zeiss s.p.a.). Images were acquired at an accelerating voltage of 5 kV and a probe current of 54 pA, using a secondary electron detector.

Atomic force microscopy

Samples for Atomic Force Microscopy (AFM) analysis were prepared following the procedure described in [pia2]. A volume of 3 µL of G4 2TBDPS aqueous solution (prepared in 150 µL of H_2O) was deposited by drop casting onto a freshly cleaved muscovite mica substrate. After three minutes of interaction with the mica surface, the sample was rinsed and subsequently dried under a fume hood. Topographic measurements were performed using an XE-70 Atomic Force Microscope (AFM) from Park Systems (Suwon, South Korea). Imaging was conducted in non-contact mode using SSS-NCHR 10M cantilevers (silicon with aluminum coating, Park Systems) with a length of 125 µm, a nominal tip radius below 5 nm, a force constant of 42 N/m, and a resonance frequency of approximately 300 kHz. Images were acquired with a resolution of 1024 × 1024 pixels at a scan frequency of 0.5 Hz per line. When necessary, AFM images were post-processed by flattening to remove background slope, and adjustments to contrast and brightness were applied. Image analysis and data processing were carried out using the XEI software provided by Park Systems.

Dynamic light scattering

The hydrodynamic size of Minisym 2TBDPS were measured by Zetasizer Nano- ZS instrument (Malvern Instrument Ltd., Cambridge, UK) equipped with a He-Ne laser (633 nm, fixed scattering angle of 173°, 25 °C). The size (d) and the polydispersity index (PDI) of the samples were obtained by dilution of 250µl Minisym 2TBDPS water solution in 1000µl MilliQ water.

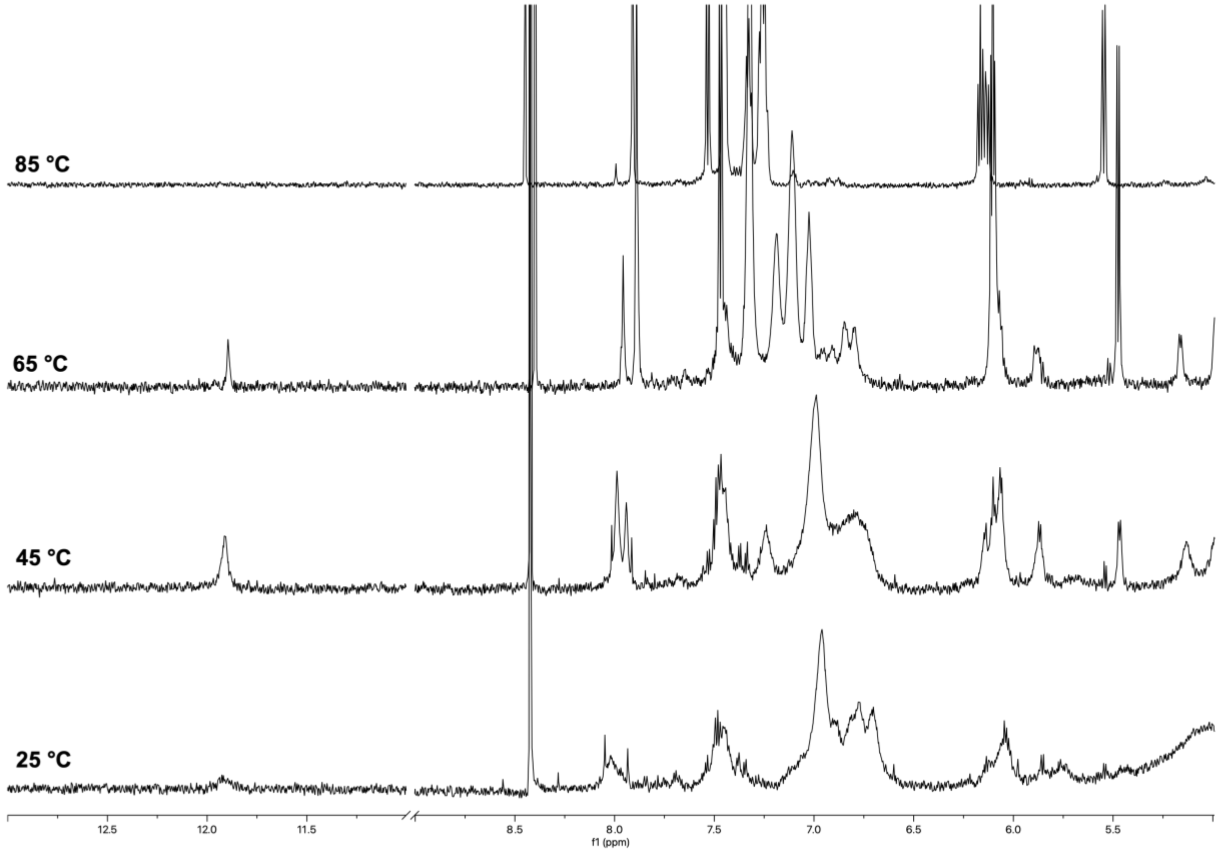

Figure S1. ^1H NMR spectra of ON **1** annealed in 20 mM K^+ buffer recorded at 25, 45, 65 and 85 °C.

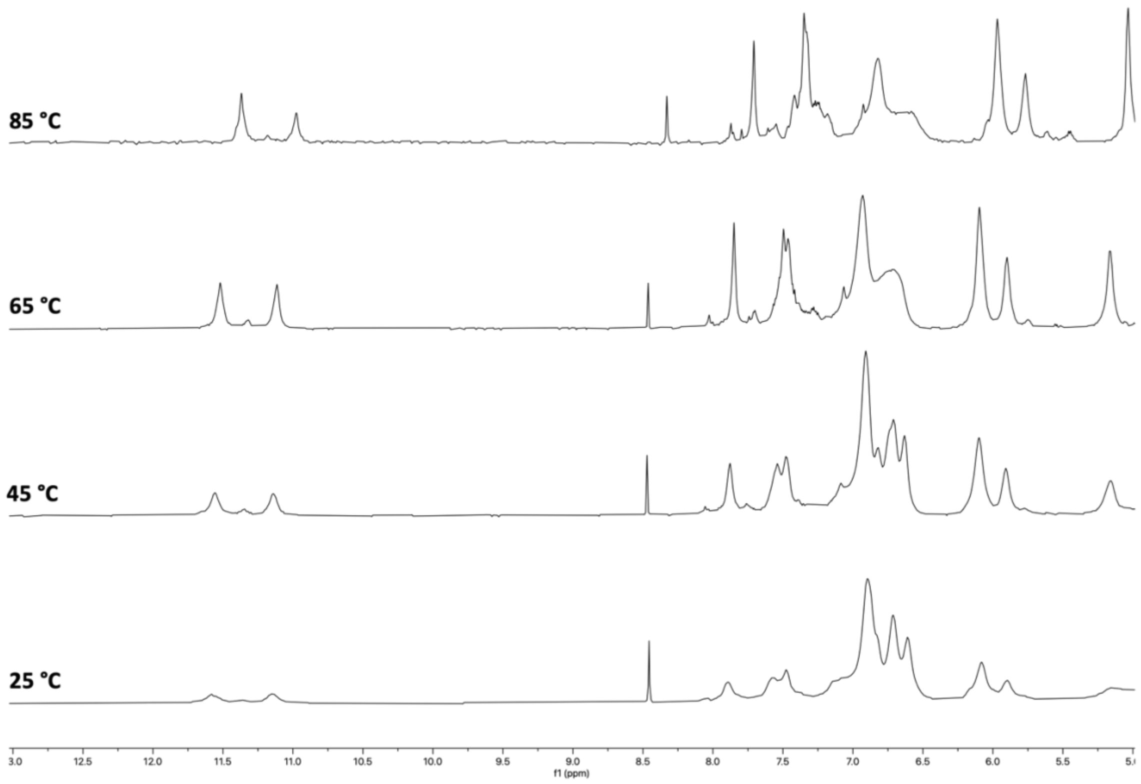

Figure S2. ^{1}H NMR spectra of ON **3** annealed in 20 mM K^{+} buffer recorded at 25, 45, 65 and 85 °C.

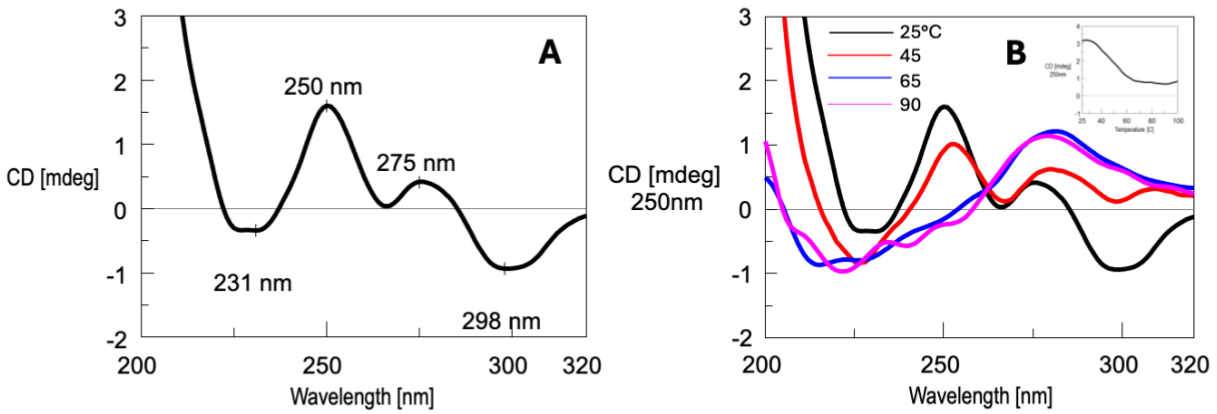

Figure S3. CD spectrum at 25 °C (A) and CD profile variation in the range 25–90 °C (B) of ON **1** annealed in a 20 mM K^+ buffer. Insert: CD melting profile at λ = 250 nm.

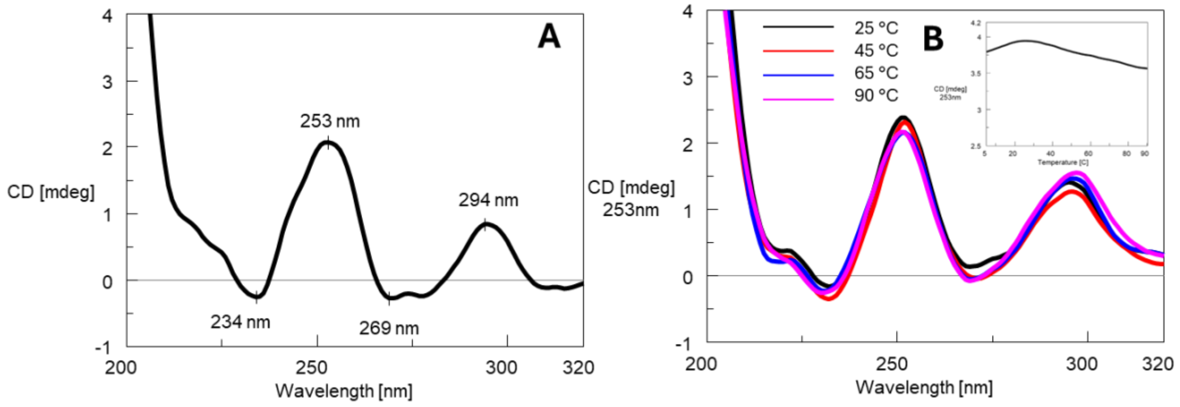

Figure S4. CD spectrum at 25 °C (A) and CD profile variation in the range 25–90 °C (B) of ON **3** annealed in a 20 mM K^+ buffer. Insert: CD melting profile at λ = 253 nm.

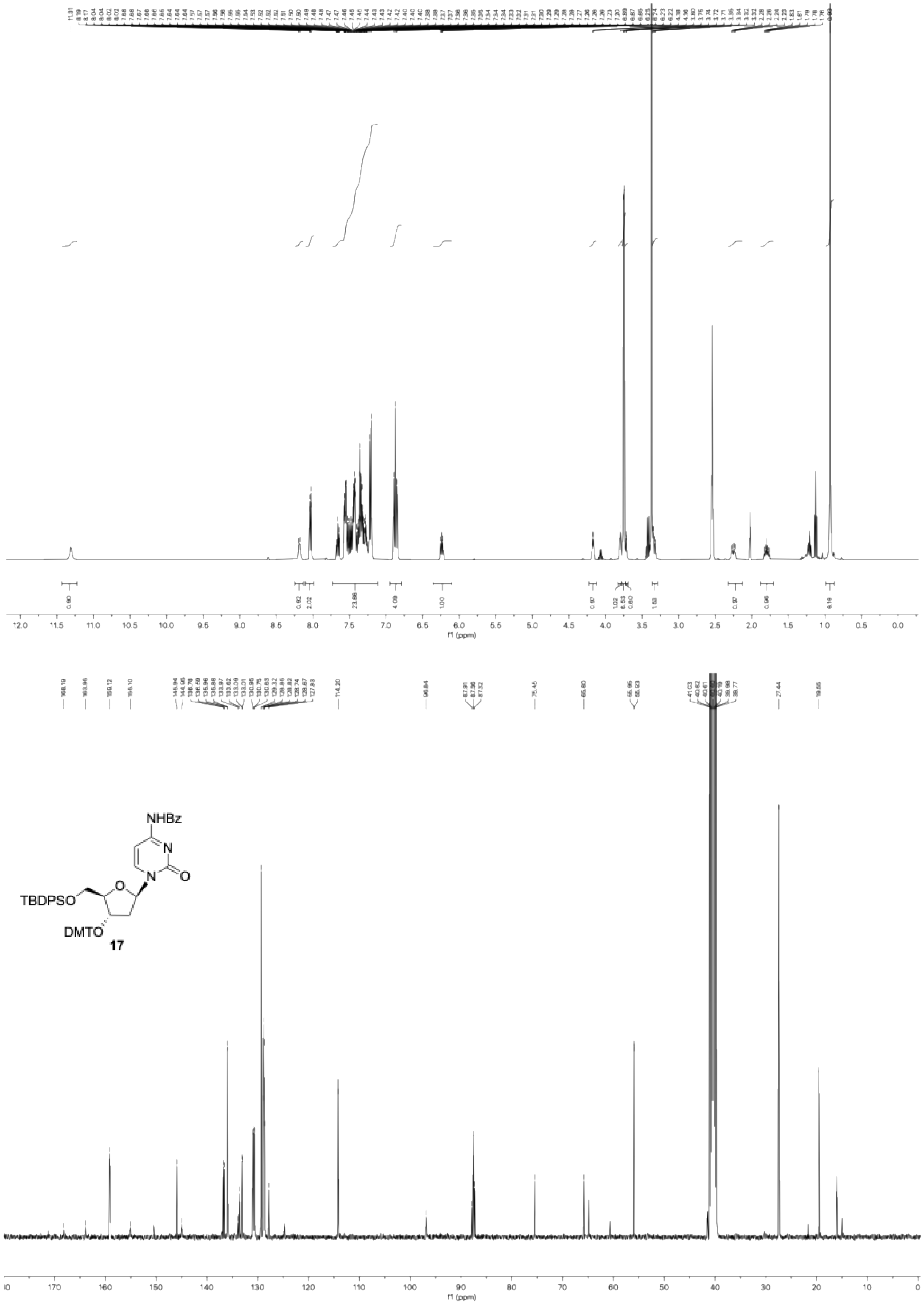

Figure S5. ^{1}H (400 MHz, DMSO-d_6) and ^{13}C NMR (101 MHz, DMSO-d_6) spectra of compound **17**.

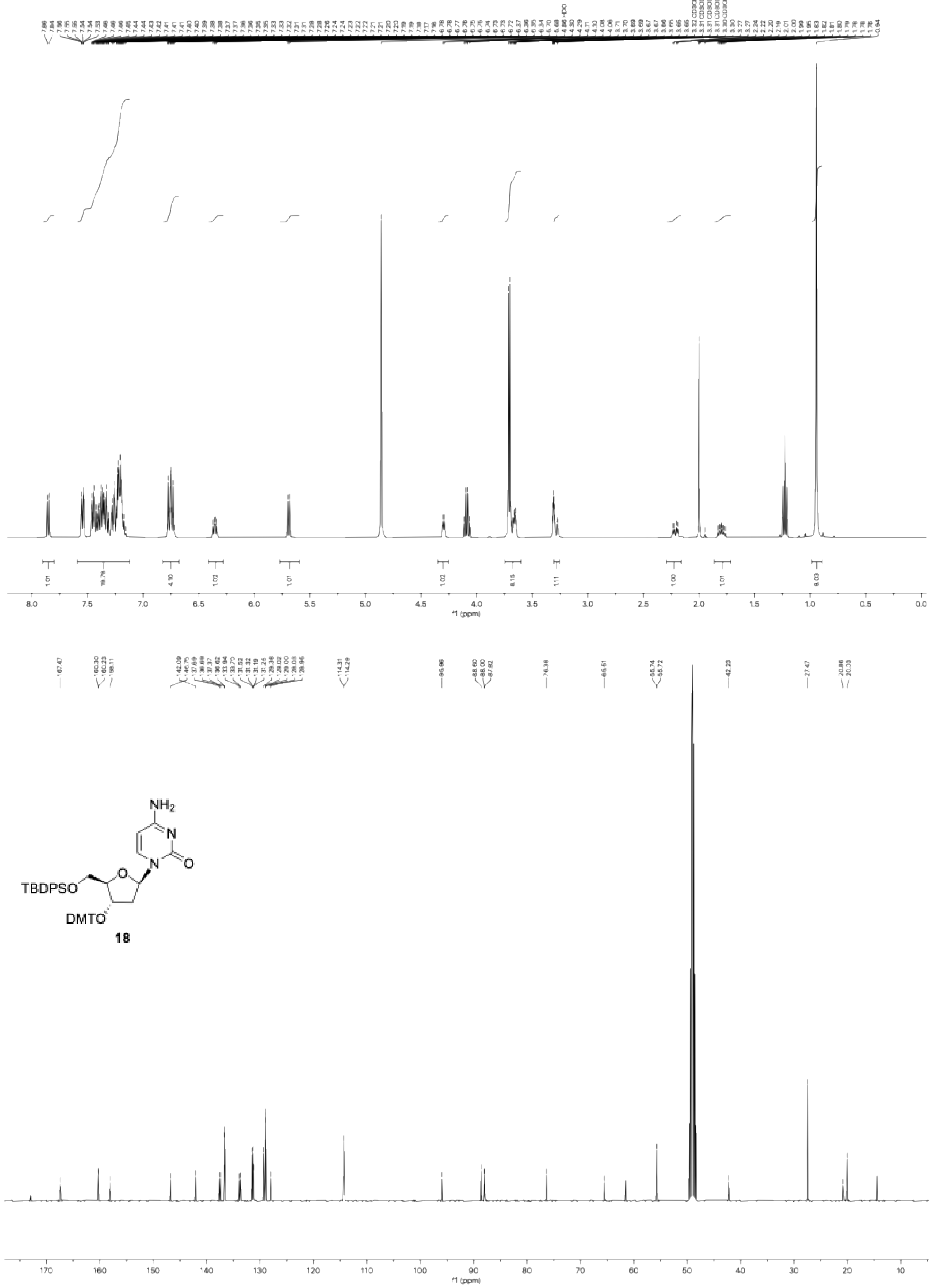

Figure S6. ^{1}H (400 MHz, CD_3OD) and ^{13}C NMR (101 MHz, CD_3OD) spectra of compound **18**.

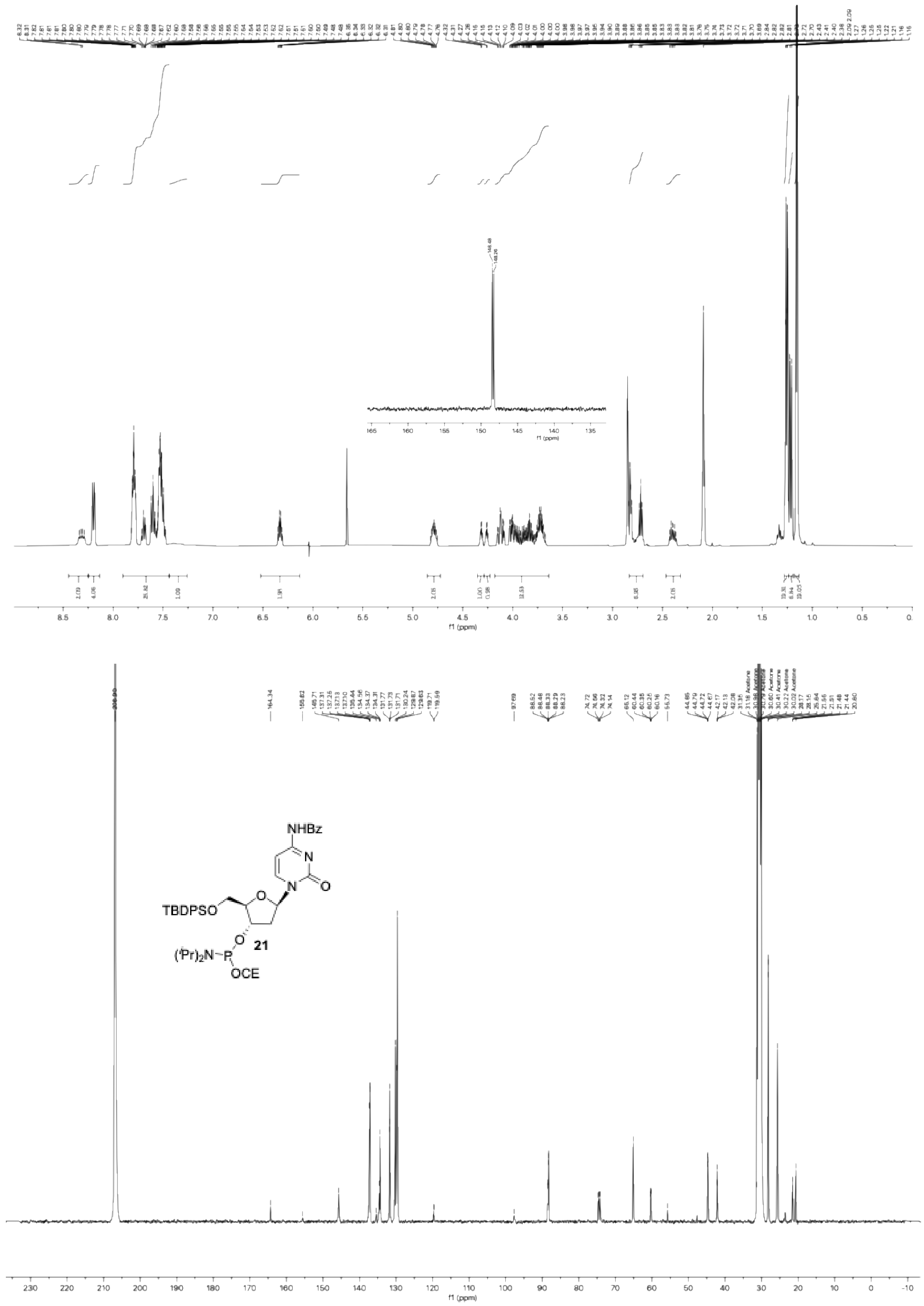

Figure S7. ^{1}H (400 MHz, Acetone-d_6), ^{31}P (162 MHz, Acetone-d_6) and ^{13}C NMR (101 MHz, Acetone-d_6) spectra of compound **21**.

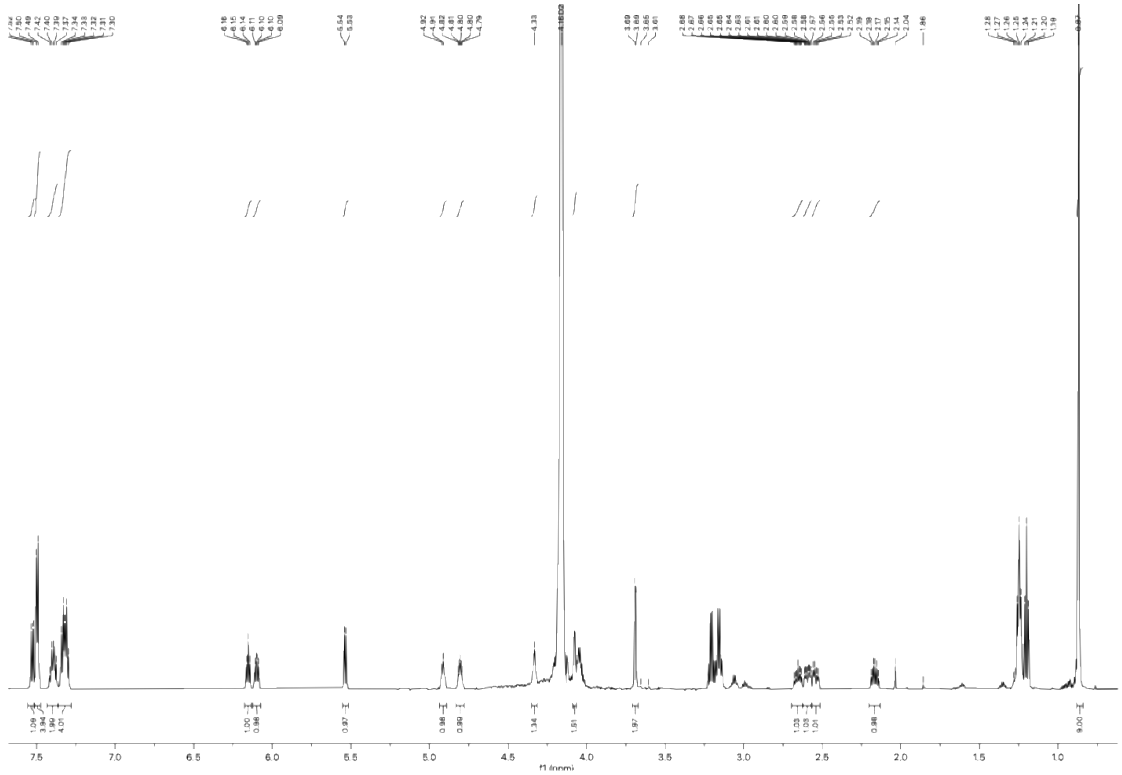

Figure S8. ^{1}H NMR (400 MHz, D_2O, 85 °C) spectrum of ON **1**.

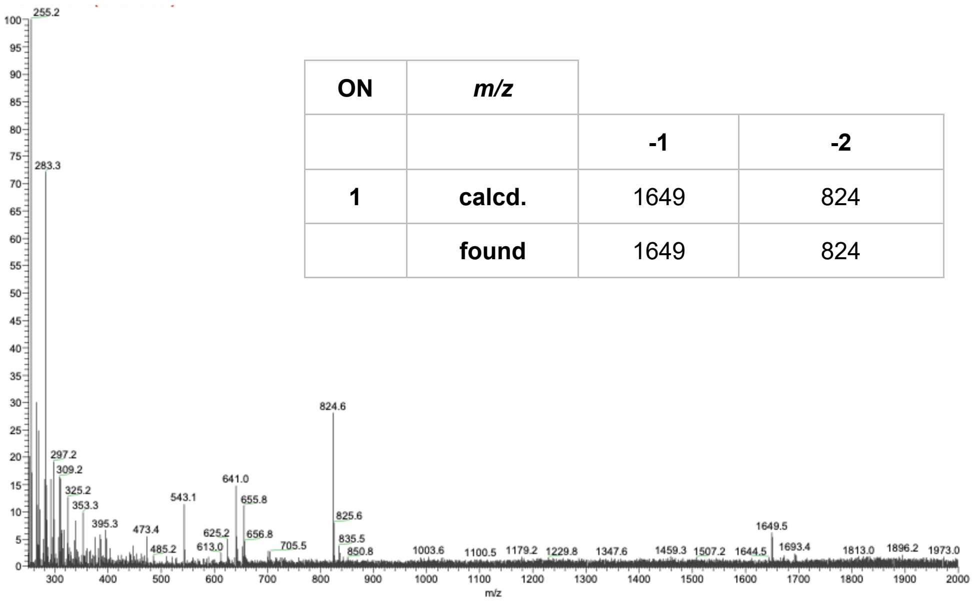

ON	*m/z*		
		-1	-2
1	calcd.	1649	824
	found	1649	824

Figure S9. ESI MS(–) spectrum of ON **1**.

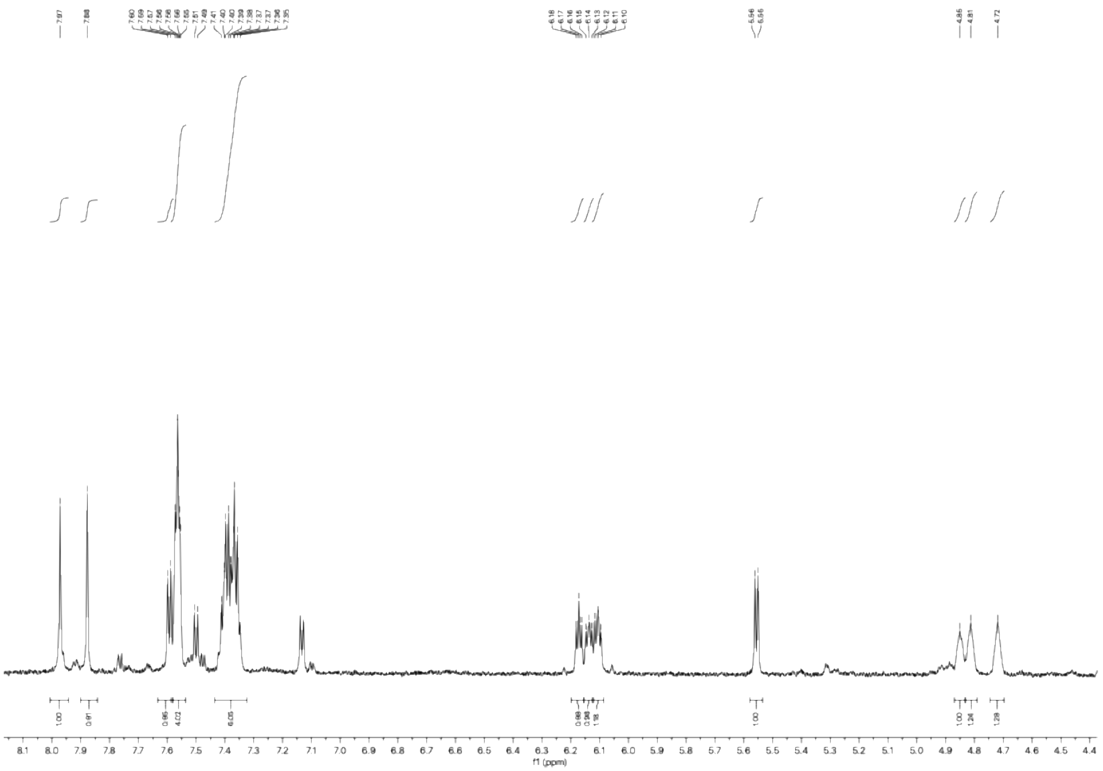

Figure S10. Expansion of ^{1}H NMR (700 MHz, DMSO-d_6/D_2O, 9:1, 45 °C) spectrum of ON **3**.

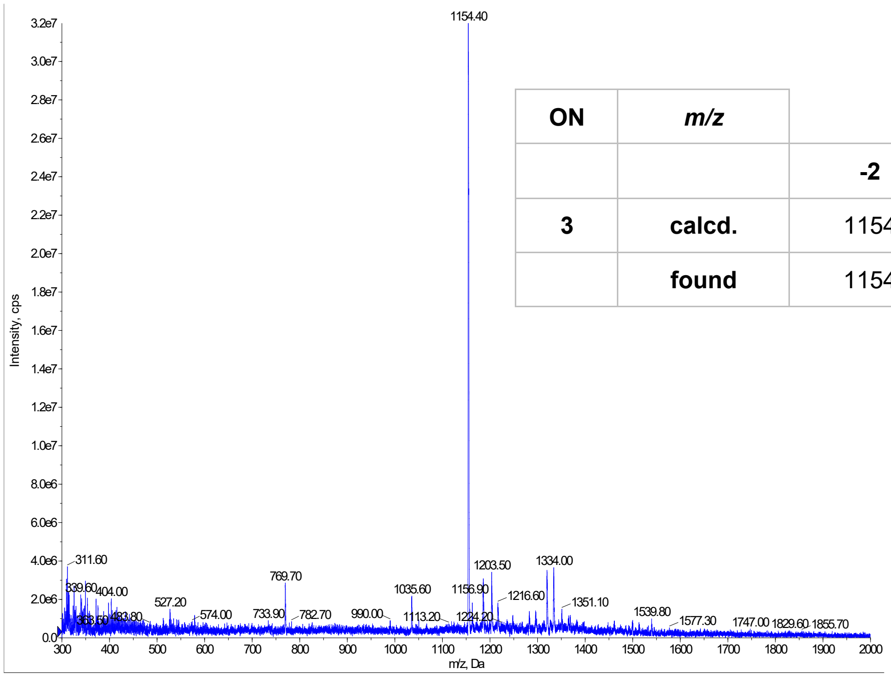

ON	*m/z*		
		-2	-3
3	calcd.	1154	769
	found	1154	769

Figure S11. ESI MS(–) spectrum of ON **3**.

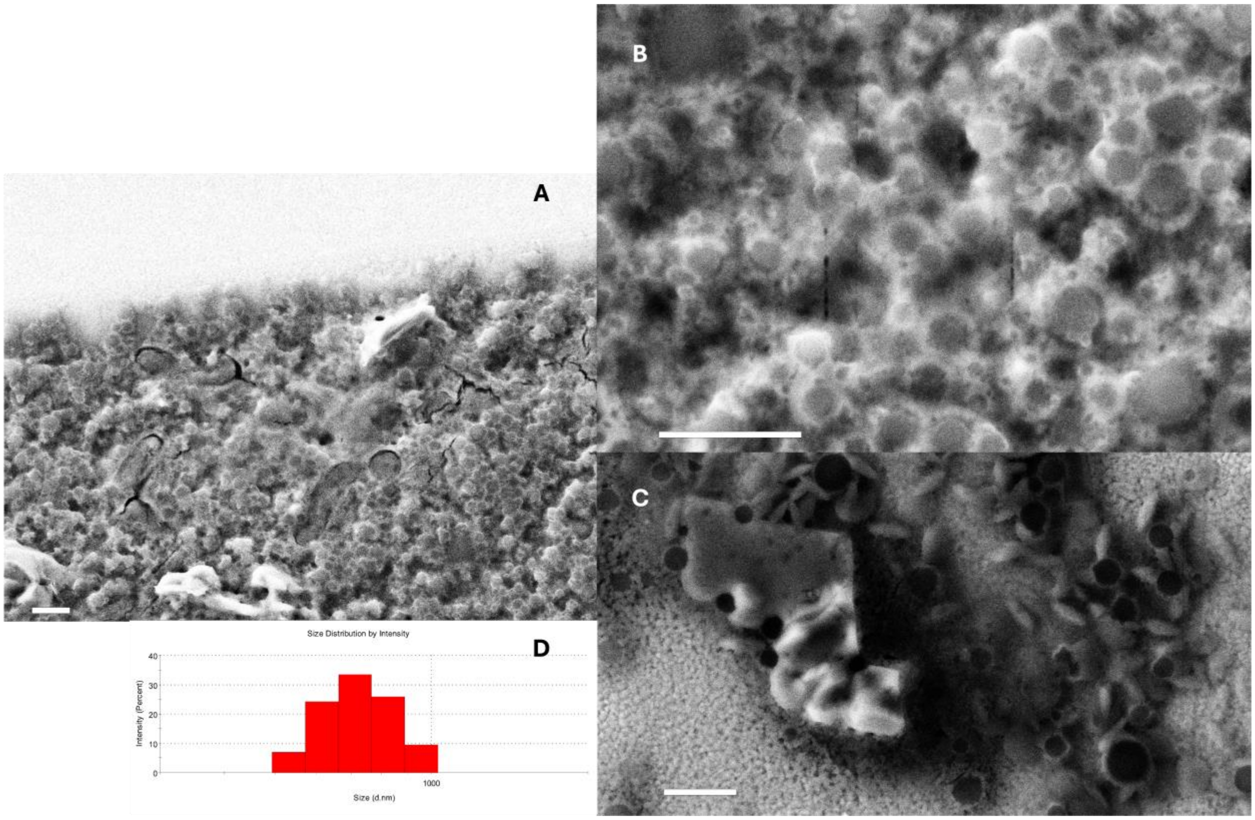

Figure S12. SEM images of G4 **2** aggregates (A-C). The scale bar in all panels is 1µm. Panels A and B display particles deposited along the periphery of the ring-shaped stain formed during the evaporation of the solvent containing the suspended material. Panel C shows particle halos located in the central region of the evaporated drop. Panel D shows the size distribution resulting from DLS measurements.
